# Disease Phenotypes and Mechanisms of iPSC-Derived Cardiomyocytes From Brugada Syndrome Patients With a Loss-of-Function SCN5A Mutation

**DOI:** 10.3389/fcell.2020.592893

**Published:** 2020-10-22

**Authors:** Wener Li, Michael Stauske, Xiaojing Luo, Stefan Wagner, Meike Vollrath, Carola S. Mehnert, Mario Schubert, Lukas Cyganek, Simin Chen, Sayed-Mohammad Hasheminasab, Gerald Wulf, Ali El-Armouche, Lars S. Maier, Gerd Hasenfuss, Kaomei Guan

**Affiliations:** ^1^Institute of Pharmacology and Toxicology, Technische Universität Dresden, Dresden, Germany; ^2^Department of Cardiology and Pneumology, University Medical Center Göttingen, Göttingen, Germany; ^3^German Center for Cardiovascular Research (DZHK), Partner site Göttingen, Göttingen, Germany; ^4^Department of Hematology and Oncology, University Medical Center Göttingen, Göttingen, Germany; ^5^Department of Dermatology, Venereology and Allergy, Charité – Universitätsmedizin Berlin, Berlin, Germany; ^6^CCU Translational Radiation Oncology, German Cancer Consortium Core-Center Heidelberg, National Center for Tumor Diseases, Heidelberg University Hospital (UKHD) and German Cancer Research Center (DKFZ), Heidelberg, Germany; ^7^Clinic for Internal Medicine II, University Hospital Regensburg, Regensburg, Germany

**Keywords:** Brugada syndrome, disease modeling, induced pluripotent stem cells, *SCN5A* mutation, depolarization, repolarization

## Abstract

Brugada syndrome (BrS) is one of the major causes of sudden cardiac death in young people, while the underlying mechanisms are not completely understood. Here, we investigated the pathophysiological phenotypes and mechanisms using induced pluripotent stem cell (iPSC)-derived cardiomyocytes (CMs) from two BrS patients (BrS-CMs) carrying a heterozygous *SCN5A* mutation p.S1812X. Compared to CMs derived from healthy controls (Ctrl-CMs), BrS-CMs displayed a 50% reduction of *I*_Na_ density, a 69.5% reduction of Na_V_1.5 expression, and the impaired localization of Na_V_1.5 and connexin 43 (Cx43) at the cell surface. BrS-CMs exhibited reduced action potential (AP) upstroke velocity and conduction slowing. The *I*_to_ in BrS-CMs was significantly augmented, and the *I*_CaL_ window current probability was increased. Our data indicate that the electrophysiological mechanisms underlying arrhythmia in BrS-CMs may involve both depolarization and repolarization disorders. Cilostazol and milrinone showed dramatic inhibitions of *I*_to_ in BrS-CMs and alleviated the arrhythmic activity, suggesting their therapeutic potential for BrS patients.

## Introduction

Brugada syndrome (BrS), a genetic heart disease, is one of the major causes of sudden cardiac death in young people. The diagnosis of BrS is based on the changes in the electrocardiogram (ECG) with a transient or persistent ST-segment elevation in the right precordial leads and the right bundle branch block (Antzelevitch and Fish, 2006). In the past two decades, extensive research on BrS has revealed parts of its genetic basis. To date, 293 different mutations in *SCN5A* encoding the α-subunit of the cardiac sodium channel (Na_V_1.5) have been implicated as possible causes of BrS (Kapplinger et al., 2010). The implantation of an automatic implantable cardiac defibrillator (ICD) or the ablation of the arrhythmogenic substrate in the right ventricular outflow tract area have been proven effective in treating ventricular tachycardia and fibrillation and preventing sudden cardiac death in BrS patients. In addition, drug therapy with low-dose quinidine has been shown to prevent arrhythmic events in BrS patients after arrhythmic storm and should be considered according to current guidelines (class IIa indication). However, prophylactic use in asymptomatic patients is controversially discussed and worldwide commercial availability is limited. Thus, a detailed understanding of arrhythmic mechanisms in BrS is required to develop novel drug therapies that are urgently needed.

For more than a decade, the underlying pathophysiological mechanism of BrS has been a matter of debate between the depolarization versus repolarization hypotheses (Wilde et al., 2010). The depolarization theory relies on right ventricular conduction slowing and involvement of structural abnormalities including increased epicardial and interstitial fibrosis, and the reduced gap junction expression (Nademanee et al., 2015). The repolarization theory is mainly supported by the late fractionated low-voltage potentials found in right ventricle epicardium but not in endocardium by using canine heart wedge model, which are associated with repolarization defects (Szel and Antzelevitch, 2014; Patocskai et al., 2017). Nonetheless, there is a general consensus that *SCN5A* mutations resulting in Na_V_1.5 loss-of-function either by the decreased expression of functional Na_V_1.5 at the sarcolemma and/or by the altered channel gating properties lead to a diminution of *I*_Na_ and contribute to the development of arrhythmias in BrS (Patocskai et al., 2017). Recently, single cardiomyocytes (CMs) derived from patient-specific and genome-edited induced pluripotent stem cells (iPSCs) were used to recapitulate the phenotypes of patients with BrS and to study the underlying mechanism (Liang et al., 2016; Veerman et al., 2016; Ma et al., 2018). iPSC-CMs from BrS patients who tested negative for mutations in the known BrS-associated genes do not exhibit clear cellular electrophysiological abnormalities (Veerman et al., 2016). Patient-specific iPSC-CMs with a double missense mutation (p.R620H and p.R811H) or with a deletion (Δ1397) in *SCN5A* could recapitulate single-cell phenotype features of BrS, including the blunted *I*_Na_, an increased triggered activity, and an abnormal Ca^2+^ handling (Liang et al., 2016).

Interestingly, a more recent study reported a remarked lower *I*_Na_ density and an increased phase-1 repolarization at a slow pacing frequency (0.1 Hz) in BrS patient-specific iPSC-CMs with a compound *SCN5A* mutation (p.A226V and p.R1629X), leading to pro-arrhythmic action potential (AP) morphology (Ma et al., 2018). The dominant repolarizing currents in human CMs are the transient-outward potassium current (*I*_to_) conducted by K_V_4.2 and K_V_4.3 channels which contribute to *I*_to,fast_, and by K_V_1.4 which accounts for *I*_to,slow_ and the rapidly and slowly activating delayed rectifier potassium currents (*I*_Kr_ and *I*_Ks_) conducted by HERG and K_V_LQT1/mink channels, respectively (Nerbonne and Kass, 2005). Previous study identified gain-of-function mutations in *KCND3* encoding K_V_4.3 in patients with BrS as a result of the increased *I*_to_ (Giudicessi et al., 2011). These studies suggest that the underlying mechanism for BrS might be different in BrS patients with different mutations and both depolarization and repolarization mechanisms might coexist.

In the present study, we utilized iPSC-CMs from two BrS patients (BrS1-CMs and BrS2-CMs) with a heterozygous *SCN5A* point mutation p.S1812X causing a premature termination codon. We show that BrS-CMs from both patients reveal a reduced *I*_Na_ and a delayed sodium channel activation, which result in a slowed AP upstroke velocity and a reduced field potential (FP) conduction velocity (CV), similar to the phenotype observed in BrS patients with *SCN5A* loss-of-function mutations. Importantly, BrS-CMs also exhibited the enhanced *I*_to_ and an augmented *I_Ca__L_* (L-type calcium current) window current, which may contribute to the arrhythmic phenotype. Exposure of BrS-CMs to cilostazol and milrinone, two clinically used phosphodiesterase (PDE) blockers, lowered *I*_to_ and alleviated arrhythmic activity, without affecting on the CV. These data indicate that the electrophysiological mechanisms underlying arrhythmia in BrS-CMs may involve an impaired coordination of *I*_Na_, *I*_to_, and *I_Ca__L_* and cilostazol and milrinone can reduce the arrhythmia but have no effect on the CV.

## Materials and Methods

### Generation of iPSCs

The study was approved by the Institutional Ethics Committee of University Medical Center Göttingen (approval number 21/1/11) and of Technical University of Dresden (approval number EK 422092019) and carried out in accordance with the approved guidelines. Bone marrow aspirate (10 mL) and peripheral blood (15 mL) were taken from the BrS1 and BrS2 patients, respectively, after obtaining written informed consent. For the Ctrl1, bone marrow aspirate of a 45-year-old female (without known cardiac disease) left over from diagnostic purposes was used. For the Ctrl2, skin biopsy was taken. Bone marrow-derived mesenchymal stem cells, peripheral blood mononuclear cells, and skin fibroblasts were cultured and reprogrammed into iPSCs using the STEMCCA lentivirus (BrS1-iPSCs, Ctrl1-iPSCs, Ctrl2-iPSCs) or Sendai virus system (BrS2-iPSCs), as described previously (Streckfuss-Bomeke et al., 2013; Cyganek et al., 2018). The generated iPSCs from the BrS patients and the healthy control were adapted to feeder-free culture conditions and cultivated on tissue culture plates pre-coated with Geltrex^®^ (Thermo Fisher Scientific) in Essential 8^TM^ medium (Thermo Fisher Scientific).

### Karyotype Analysis

Karyotyping of BrS- and Ctrl-iPSCs was performed using standard methodology. Prior to karyotyping, the iPSCs were cultured on feeder-free conditions. The cells were treated with 100 ng/mL colcemid (Thermo Fisher Scientific) for 16 h. The metaphases were prepared according to standard methodology and subjected to Giemsa staining before analysis with a light microscope (Zeiss Axio Imager.M2). The karyotype was analyzed and documented by using the software Case Data Manager 6.0 (Applied Spectral Imaging).

### Genomic Sequencing

The genomic DNA of Ctrl- and BrS-iPSCs was isolated and purified using the automated Maxwell^®^ 16 cell DNA purification kit (Promega) according to the manufacturer’s instructions. DNA sequence comprising the *SCN5A* point mutation site in the generated Ctrl- and BrS-iPSCs was amplified by PCR. The primer set used is given in Supplementary Table 3. The PCR products were purified using the QIAquick^®^ gel extraction kit (Qiagen) according to the manufacturer’s instructions and sequenced by a commercial sequencing facility (Seqlab, Göttingen).

### Spontaneous *in vitro* Differentiation

For spontaneous *in vitro* differentiation experiments, the cells were digested with 200 U/mL collagenase type 4 (Worthington Biochemicals) into big clusters and cultivated in suspension to form multicellular aggregates, known as embryoid bodies (EBs) for 8 days. The differentiation medium contained IMDM (Thermo Fisher Scientific), 20% fetal bovine serum, 1x non-essential amino acid (Thermo Fisher Scientific), and monothioglycerol (450 μM, Sigma–Aldrich). EBs were thereafter plated onto 0.1% gelatin-coated tissue culture dishes, cultivated up to 1 month and used for further analyses.

### Reverse Transcriptase-PCR (RT-PCR) and Quantitative Real-Time PCR

BrS1- and BrS2-iPSCs and Ctrl1-iPSCs were characterized for the expression of pluripotency genes *NANO*G, *LIN28*, *GDF3*, and *FOXD3* using reverse transcriptase-PCR (RT-PCR). Expression of tissue-specific genes *ALB* (albumin), α*-MHC* (myosin heavy chain), *TNNT2* [cardiac troponin T (cTnT)], and *TH* (thyroxine hydroxylase) was analyzed during EB differentiation. To assess the expression of *KCND2/3* and *KCNA4* genes, 3-month-old CMs derived from Ctrl- and BrS-iPSCs were collected as cell pellets for further use.

Total mRNA was isolated as described in the SV Total RNA Isolation System with on-column DNase digestion (Promega). DNase-treated RNA (200 ng) was used for the first-strand cDNA synthesis by using Murine Leukemia Virus Reverse Transcriptase and Oligo d(T)_16_ (Thermo Fisher Scientific). One-tenth of the cDNA reaction was taken as PCR template and amplified for 25–35 cycles, denaturation at 95°C for 15 s, annealing at 52–64°C for 15–30 s depending on the primer melting temperatures, and elongation at 72°C for 30 s. The primer sequences (forward and reverse), annealing temperatures, and the cycles used for RT-PCR analyses are listed in Supplementary Table 3.

Quantitative real-time PCR were carried out using SsoAdvanced Universal SYBR Green PCR super mix, Hard-Shell Optical 96-well plates, and the BIO-RAD CFX96 Real-time PCR system (Bio-Rad) according to the manufacturer’s instructions. Initial denaturation was performed at 95°C for 30 s, followed by 45 amplification cycles of 15 s at 95°C and 1 min elongation at 60°C. A melting curve was obtained for 65–95°C. PCR products were confirmed by the melting temperature and agarose gel electrophoresis. Expression was quantified using ΔΔCt method with *RPL32* as a reference. Sequences for *RPL32*, *TNNT2*, *KCND2/3*, and *KCNA4* real-time PCR analyses are listed in Supplementary Table 3.

### Teratoma Formation Analysis

To investigate their differentiation potential, the generated iPSCs were differentiated into derivatives of the three germ layers *in vivo* as previously described (Streckfuss-Bomeke et al., 2013). Briefly, the undifferentiated iPSCs were subcutaneously injected into 8-week-old recombination activating gene 2 and gamma C deficient (RAGC) mice. The mice were sacrificed 8–12 weeks after injection and the resulting teratomas were examined with hematoxylin and eosin staining.

### Directed Differentiation of iPSCs Into CMs

All iPSCs were cultured on Geltrex^®^ in Essential 8^TM^ medium as a monolayer with 80–100% confluence before the initiation of differentiation. At day 0, cells were cultured in RPMI1640 medium (Thermo Fisher Scientific) with Glutamax and HEPES (Thermo Fisher Scientific), 0.5 mg/mL human recombinant albumin, and 0.2 mg/mL L-ascorbic acid 2-phosphate and treated with 4 μM CHIR99021 (Merck Millipore), a highly selective inhibitor of GSK3β. After 48 h, CHIR99021 was removed and the cells were treated with 5 μM Wnt antagonist II (IWP2, Merck Millipore). Another 48 h later, the medium was replaced without IWP2. From day 8 on, the medium was replaced by RPMI1640 with Glutamax and HEPES containing 1x B27 supplement with insulin (Thermo Fisher Scientific). First beating CMs appeared around day 8. At day 25, the CMs were first treated with collagenase 2 (200 U/mL, Worthington) for 30 min so that beating clusters could be detached from the culture dish. These clusters were collected and digested with 0.25% trypsin/EDTA solution (Thermo Fisher Scientific) into single cells, which were replated onto Geltrex^®^ -coated culture dishes as a monolayer. CMs were kept in culture for 3 months for maturation and further electrophysiological and gene and protein expression analyses.

### Allele-Specific Expression Analysis of the *SCN5A* Gene

For the allele-specific expression analysis of *SCN5A* in BrS-CMs, the mRNA was sequenced with primers containing specific barcodes using the Ion Torrent semiconductor sequencing system (Thermo Fisher Scientific). The genomic DNA was used as control.

Total mRNA was isolated and reverse transcribed into cDNA. The primer set (forward: 5’-GAG AGC ACC GAG CCC CTG AGT GAG G-3’ and reverse: 5’-CAC CAT GGG CAG GTC CAT GTT GAT G-3’) was used to amplify the region containing the mutation site. The PCR product was diluted 1/200 and used for a second PCR run with 12 cycles using the same forward primer and different custom-designed reverse primers (Sigma–Aldrich), which contained a unique barcode to distinguish individual samples. The DNA concentration of each sample was measured with Qubit 2.0 Fluorometer (Thermo Fisher Scientific) and 250 ng of each sample was pooled and electrophoretically separated on a 2% agar gel. The specific product was extracted from the gel in a QIAcube system using the QIAquick^®^ gel extraction kit (Qiagen) and subsequently purified with Agencourt AMPure XP PCR purification kit (Beckman Coulter) according to the manufacturer’s instructions and eluted in low TE buffer (Thermo Fisher Scientific). The DNA quantity was determined by real-time PCR analysis in a 384-well plate using the GeneRead Library Quant kit (Qiagen). 400 μL of 10 pmol PCR product was used for clonal amplification onto Ion Sphere particles accomplished by an emulsion PCR in the Ion OneTouch (Thermo Fisher Scientific) system according to the manufacturer’s instructions. The Ion Sphere particles coated with the amplified template DNA were applied to an Ion Torrent sequencing chip and placed on the Ion Personal Genome Machine (Thermo Fisher Scientific) for sequencing.

### Immunofluorescence Staining

Undifferentiated iPSCs or EB cultures at different differentiation stages as well as 3-month-old CMs were fixed with 4% paraformaldehyde (Sigma–Aldrich) in PBS at room temperature for 20 min and then blocked with 1% bovine serum albumin (Thermo Fisher Scientific) at 4°C overnight. The primary and respective secondary antibodies used are listed in Supplementary Table 4. Nuclei were counter-stained with 4-6-diamino-2-phenylindole (DAPI, 0.4 μg/mL, Sigma–Aldrich). Samples were mounted with Fluoromount-G mounting medium (eBioscience). Antibodies staining against non-transmembrane proteins were additionally treated with 0.1% Triton X-100 (Sigma–Aldrich) in PBS to permeabilize the cell membrane. Fluorescent images were captured with a fluorescence microscope (Zeiss Observer.Z1 or Axio Imager.M2). For some images, the ApoTome modus (Zeiss) was used.

### Western Blot

For analysis of protein levels, Ctrl- and BrS-CMs were solubilized in 0.5% digitonin, 1.5% NP-40, and 1.0% Triton X 100 solubilization buffer for 35 min at 4°C. Protein concentrations were determined with a BCA assay kit. Equal amounts of protein were loaded on biphasic 6 and 10% sodium dodecyl sulfate polyacrylamide gel (SDS-PAGE) and run at low voltage. After blotting to PVDF membranes, membranes were incubated with Na_V_1.5, Cx43, Ca_V_1.2, K_V_4.3, and cTnT antibodies overnight. The primary and respective secondary antibodies used for Western blots are listed in Supplementary Table 4. Western blots were developed with a peqlab Fusion FX Vilber Lourmat camera and analyzed using the FusionCapt Advance 16.12 software.

### Patch-Clamp

Whole-cell clamp was used to measure membrane potential (current-clamp configuration), *I*_Na_, late *I*_Na_, *I*_to_, and *I*_CaL_ (voltage-clamp configuration), as previously described (Wagner et al., 2006; Streckfuss-Bomeke et al., 2013). All experiments were conducted with 3-month-old Ctrl- and BrS-CMs at room temperature. The pipette and extracellular solutions for various kinds of voltage and current patch-clamp recordings are listed in Supplementary Table 5.

Spontaneous APs were recorded immediately after rupture with the EPC10 amplifier (HEKA Elektronik) using the Patchmaster software (HEKA Elektronik). No current was injected into the cells. Signals were filtered with 2.9 and 10 kHz Bessel filters. At least 20 spontaneous APs in a row were analyzed and averaged using LabChart Pro software (ADInstruments) to determine *V*_max_, APA, and RMP.

For current measurements, all recordings started at least 1 min after rupture with the EPC10 amplifier using the Patchmaster software. Signals were filtered with 2.9 and 10 kHz Bessel filters and recorded with an EPC10 amplifier. Membrane capacitance (C_m_) and series resistance (R_s_) were compensated automatically after rupture, and currents were normalized to C_m_.

The pulse stimulation of sodium current recording was picturized in inset of Figures 3B,D–F. The current–voltage (*I*–*V*) relationship was determined by increasing the voltage stepwise from −95 mV to +5 mV in 5 mV steps at a holding potential of −100 mV. Each pulse lasted 50 ms. Steady-state inactivation was measured by a two-pulse protocol at a holding potential of −100 mV. The voltage of the first conditioning pulse (500 ms) was increased stepwise from −120 to −20 mV in 5 mV steps. The second pulse to −20 mV was used to measure *I*_Na_ and normalized to the maximum *I*_Na_ (which was usually after the first pulse). For the measurement of the recovery from inactivation of sodium channels, a two-pulse protocol was used, with an increasing delay between the two pulses ranging from 1 to 165 ms. The first conditioning pulse (1000 ms) is used to induce sodium channel inactivation and the second pulse is used to measure *I*_Na_ generated by sodium channels that recover from inactivation. The *I*_Na_ from the second pulse was normalized to the maximal *I*_Na_ initiated by the conditioning pulse.

To study the effect of milrinone on *I*_Na_ in Ctrl- and BrS-CMs, an automated patch-clamp system (Patchliner Quattro, Nanion Technologies GmbH) was used with low resistance NPC-16 chips and *I*_Na_ was recorded at room temperature. iPSC-CMs were dissociated into single cells for automated patch-clamp analysis, as previously described (Li et al., 2019). The liquid junction potentials and series resistance were not compensated. The holding potential was −100 mV. *I*_Na_ was recorded using voltage steps from −90 to +70 mV for 20 ms in 5 mV steps at an interval of 2000 ms. Currents were sampled at 25 kHz and low-pass-filtered at 2.9 kHz.

The pulse protocols for *I*_to_ current were shown in inset of Figures 4B,C,F. The current–voltage (*I*–*V*) relationship was determined by increasing the voltage stepwise from −40 to +60 mV in 10 mV steps at a holding potential of −90 mV with a 20 ms prepulse at −35 mV to inactivate sodium channel. Each pulse lasted 600 ms. Steady-state inactivation was measured by using a two-pulse protocol at a holding potential of −90 mV. The voltage of the first conditioning pulse (600 ms) was increased stepwise from −90 to +40 mV in 10 mV steps after a 20 ms prepulse at −35 mV to inactivate sodium channels. The second pulse of 600 ms at +60 mV was used to measure *I*_to_ and normalized to the maximum *I*_to_. For the measurement of the recovery from inactivation of *I*_to_, a two-pulse protocol was used, with an increasing delay between the two pulses ranging from 0 to 10,240 ms. The pulse was depolarized from −90 to +40 mV and last for 600 ms. The *I*_to_ from the second pulse was normalized to the maximal *I*_to_ initiated by the conditioning pulse. The pulse intervals were 15 s for all the stimulations.

The impulse protocol for *I*_CaL_ was shown in inset of Figures 4H,I,K, as previously described (Luo et al., 2020). The current–voltage (*I*–*V*) impulse depolarized from −90 to −80 mV then every 10 mV as step to +70 mV with a duration of 600 ms. Steady-state inactivation was measured by using a two-pulse protocol at a holding potential of −90 mV. The voltage of the first conditioning pulse (600 ms) was increased stepwise from −90 to +10 mV in 10 mV steps. The second pulse of 600 ms at 0 mV was used to measure *I*_CaL_ and normalized to the maximum *I*_CaL_. For the measurement of the recovery from inactivation of *I*_CaL_, a two-pulse protocol was used, with an increasing delay between the two pulses ranging from 0 to 1460 ms. The pulse was depolarized from −90 to 0 mV and last for 600 ms. The *I*_CaL_ from the second pulse was normalized to the maximal *I*_CaL_ initiated by the conditioning pulse. The pulse intervals were 3 s for all the stimulations.

For the conductance (*G*/*G*_max_) calculation, a custom built-in formula and add-in modular in excel were used. The same fit functions were used for analyzing the kinetics of *I*_Na_ and *I*_CaL_. Steady-state activation and inactivation curves were fitted with a standard Boltzmann function: *Y* = 1/[1 + exp((*V*_1__/__2_ – *V*)/*k*_∞_)], where *V*_1__/__2_ is the half-maximal voltage of steady-state (in)activation and *k*_∞_ is the slope factor of the voltage dependence of (in)activation. The development of the recovery from inactivation was fitted with single exponential function: *Y*(*t*) = *Y*_0_ + *A*(1 – exp(−*t*/*τ_rec_*)), where *A* is the amplitude and *τ_rec_* the time constant of recovery from inactivation. Persistent *I*_Na_ was measured by integrating the current between 50 and 450 ms of a 1000 ms pulse from a holding potential of −100 to −20 mV. The probability of *I*_CaL_ window current was calculated from steady-state (in)activation parameters through the following equation as previously described: Probability = 1/{1 + exp[(*V*_1__/__2_*_,act_* − *V*)/*k*_act_]} ^∗^ (1/{1 + exp[(*V* − *V*_1__/__2_*_,inact_*)/*k*_inact_]}) (Huang et al., 2011).

The data were analyzed using Clampfit (Axon Instruments), Fitmaster (HEKA Elektronik), and GraphPad Prism (GraphPad Software, Inc.) software.

### Multielectrode Array

The digested iPSC-CMs were seeded on Geltrex^®^ -coated MEA chips. The final concentration was approximately 200,000 iPSC-CMs in 50 μL RPMI1640 medium supplemented with 1x B27. The medium was replenished to 1 mL after seeding for around 30 min. In this study, 60MEA200/30iR-Ti-gr chips were used (Multichannel Systems). The medium was changed one day after digestion and thereafter every two days until day 6, which performed the recording. All the recordings were carried out at 35–37°C by using Cardio 2D software (Multichannel Systems). The sample rate was 10,000 Hz. The spontaneous beating frequency and inter-beat interval were automatically generated by Cardio 2D. At least five stable continuous CV values were averaged from spontaneous beatings. For FP metrics [peak-to-peak amplitude (PPA), peak-to-peak duration (PPD), and peak-to-peak slope (PPS)] analysis, the averaged measurements (3 min) of the electrodes (Pin numbers: 22, 33, 44, 55, 66, and 77) were analyzed by Cardio 2D+ software. The rate of arrhythmia was quantified as standard deviation (SD) of the inter-beat interval, which was normalized to averaged inter-beat intervals.

### Statistics

Data are presented as the mean ± standard error of the mean (SEM). Statistical analysis was performed with GraphPad Prism 7 using the two-tailed unpaired Student’s *t*-test to compare differences between two independent groups, one-way ANOVA followed by Tukey’s *post hoc* test to compare more than two groups, and the paired Student’s *t*-test to compare differences between two dependent groups or the two-way ANOVA test for comparison of more groups and conditions. Results were considered statistically significant when the *P* value was <0.05 (^∗^*P* < 0.05; #*P* < 0.01; §*P* < 0.001).

## Results

### Generation and Characterization of iPSCs

We recruited two BrS patients, a 50-year-old male (BrS1) and his biological sister (BrS2) in the study. The ECG recordings of both patients at rest displayed the BrS-typical pattern, a coved-type ST-segment elevation in the right precordial leads V_1_ and V_2_ and a more saddleback pattern in V_3_ followed by a negative T wave (Supplementary Figure 1A). In addition, both patients revealed first-degree AV block. In long-term ECG recordings of patient BrS1, multiple episodes of polymorphic ventricular tachycardia appeared during stair climbing (Supplementary Figure 1B). Both patients received an ICD for the treatment. Genetic screening for possible mutations in several cardiac-specific genes (*KCND3*, *KCNQ1*, *HERG*, *KCNE1*, and *SCN5A*) revealed the heterozygous *SCN5A* point mutation C > A at position c.5435 in both patients (Schulze-Bahr et al., 2003). This non-sense mutation results in a premature termination codon causing the protein truncation at amino acid 1812 (p.S1812X). Genetic tests of their family members identified the mother as an asymptomatic carrier of the mutation, showing first-degree AV block, but no BrS-specific ECG pattern at rest. A brother died from sudden cardiac death at the age of 15. The father and another brother do not carry the mutation and show no BrS phenotype (Supplementary Figure 1C).

We generated iPSCs from these two patients (BrS1 and BrS2) by overexpression of the four Yamanaka factors *SOX2*, *KLF4*, *OCT4*, and *c-MYC* in somatic cells (Supplementary Figure 2). Three clones per individual were selected for further characterization (BrS1.1-3 and BrS2.1-3). In addition, we included control iPSC lines (Ctrl1.1-3 also known as MSC3-iPS1-3; and Ctrl2.1 and 2 also named as FB2-iPS1 and 2) from two independent healthy donors Ctrl1 and Ctrl2, which were described in our previous studies (Streckfuss-Bomeke et al., 2013; Cyganek et al., 2018). All generated BrS-iPSCs showed the typical morphology similar to Ctrl-iPSCs, were positive for pluripotency markers (Supplementary Figure 2A), showed expression of endogenous pluripotency genes (Supplementary Figure 3A), and revealed normal chromosome numbers in more than 90% of cells (Supplementary Figure 2C). Genomic sequencing confirmed the presence of the *SCN5A* mutation c.C5435A (p.S1812X) in BrS1- and BrS2-iPSCs and its absence in both Ctrl1- and Ctrl2-iPSCs (Supplementary Figure 2B). Furthermore, Ctrl- and BrS-iPSCs were differentiated into derivatives of all three embryonic germ layers *in vitro* (Supplementary Figures 3B,C) and developed teratomas in immune-deficient mice (Supplementary Figure 2D). To reduce the phenotypic variability among different cell lines, all three lines of every individual were used for the following studies.

### Reduced Na_V_1.5 and Cx43 Expression in BrS-CMs

**FIGURE 1:**
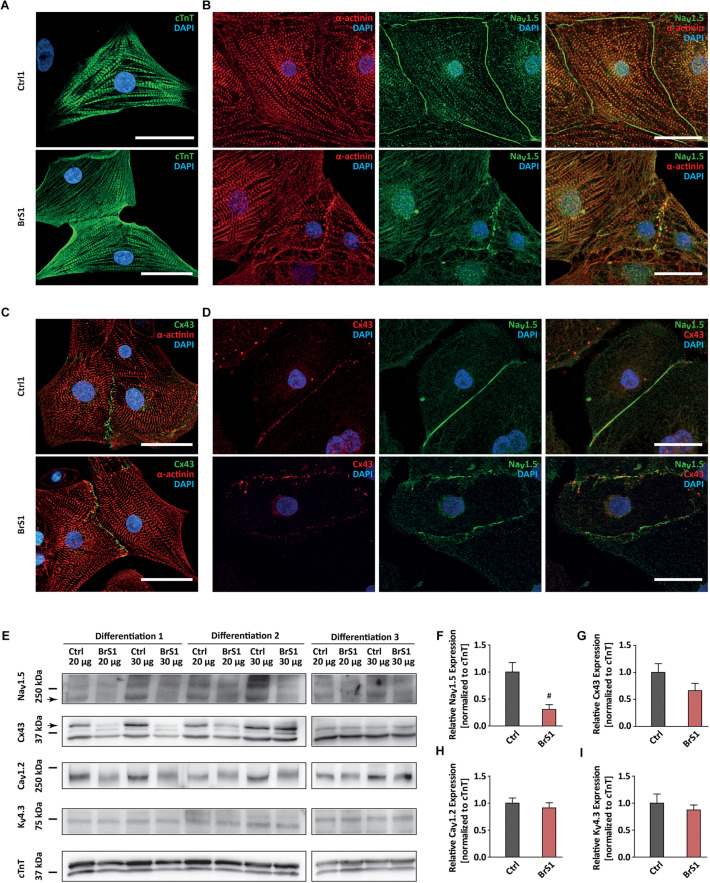
The expression of cardiac-specific proteins in Ctrl- and BrS-CMs. **(A)** Immunostaining of Ctrl- and BrS-CMs for cardiac markers cTnT. Scale bar, 20 μm. **(B)** Double-immunostaining of α-actinin and Na_V_1.5. Scale bar, 20 μm. **(C)** Double-immunostaining of Cx43 and α-actinin. Scale bar, 20 μm. **(D)** Double-immunostaining of Ctrl- and BrS-CMs with antibodies against Cx43 and Na_V_1.5. Scale bar, 20 μm. **(E)** Shown are original western blots for the decoration of Na_V_1.5 α subunit, Cx43, Ca_V_1.2, K_V_4.3, and cTnT. The arrows represent the target bands used for quantification. Quantitative analysis of Na_V_1.5 α subunit **(F)**, Cx43 **(G)**, Ca_V_1.2 **(H)**, and K_V_4.3 **(I)** expression (normalized to cTnT). Data are presented as mean ± SEM. Two-tailed unpaired Student’s *t*-test was used for statistical analysis: #*P* < 0.01.

We differentiated Ctrl- and BrS-iPSCs into ventricular CMs using the directed differentiation method, as described previously (Cyganek et al., 2018). This method gave rise to the differentiation efficiency with more than 90% of cells positive for cTnT at day 25 after the initiation of differentiation. No significant difference in cardiac differentiation efficiency was observed between Ctrl- and BrS-iPSCs (data not shown). Immunofluorescence analyses using antibodies against the cardiac-specific proteins cTnT and α-actinin revealed well-organized cross-striations in both 3-month-old BrS- and Ctrl-CMs (Figures 1A–C). To study whether the p.S1812X mutation affected the cellular localization of Na_V_1.5 in BrS-CMs, we applied an antibody that detects both full-length and truncated proteins of Na_V_1.5. We found a different distribution of Na_V_1.5 in Ctrl- and BrS-CMs (Figure 1B). Whereas equal distribution of Na_V_1.5 along cell membrane was clearly observed in Ctrl-CMs, Na_V_1.5 appeared irregular and intermittent on the cell surface. Similar to the findings of others (Malan et al., 2011; Davis et al., 2012), Na_V_1.5 was also expressed in a diffused or a fine granular-like pattern in the cytosol of both Ctrl- and BrS-CMs. In some Ctrl- and BrS-CMs, we observed that Na_V_1.5 was localized in a striated pattern, mostly overlapped with α-actinin (Figure 1B), as described in adult CMs (Mohler et al., 2004; Xi et al., 2009; Shy et al., 2014). The expression of connexin 43 (Cx43) was detected at the intercellular gap junctions in both Ctrl- and BrS-CMs (Figures 1C,D), indicating a cell-to-cell coupling. However, the co-staining of Cx43 with Na_V_1.5 revealed the strong overlap and interaction of Cx43 with Na_V_1.5 in Ctrl-CMs, but less in BrS-CMs (Figure 1D).

Western blot analyses using an antibody that only detects the full-length protein of Na_V_1.5 showed a significantly reduced expression of Na_V_1.5 in BrS-CMs by 69.5% compared to Ctrl-CMs (*P* = 0.0093; Figures 1E,F). Interestingly, Cx43 expression in BrS-CMs was also reduced by 34%, but not significantly, compared to Ctrl-CMs (*P* = 0.1443; Figures 1E,G). Furthermore, we assessed the expression of Ca_V_1.2 and K_V_4.3 in Ctrl- and BrS-CMs and did not observe significant differences between Ctrl- and BrS-CMs (Figures 1E,H,I). Analysis of the allele-specific *SCN5A* expression in BrS1- and BrS2-CMs revealed the balanced expression of *SCN5A* between the wild-type and mutated alleles (Supplementary Figure 3D).

### Conduction Slowing in BrS-CMs

**FIGURE 2:**
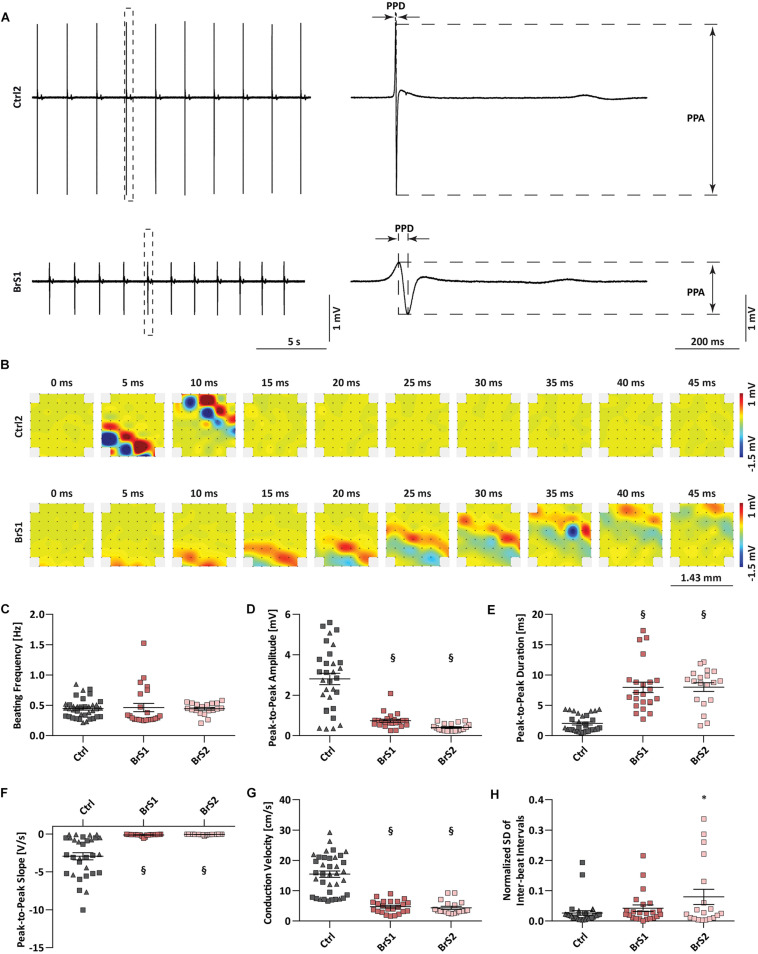
Field potential and conduction velocity of Ctrl- and BrS-CMs. **(A)** Represented original field potential traces of Ctrl2- and BrS1-CMs were enlarged to show PPA, PPD, and PPS. **(B)** Represented beats of Ctrl2- and BrS1-CMs were dissected to 10 frames (0, 5, 10, 15, 20, 25, 30, 35, 40, 45 ms), respectively. The width and length of the electrodes distributed area were 1.43 mm. The metrics of MEA measurements including beating frequency **(C)**, PPA **(D)**, PPD **(E)**, PPS **(F)**, CV **(G)**, and normalized SD of inter-beat interval **(H)** in Ctrl-CMs, BrS1-CMs, and BrS2-CMs. Gray triangle represents Ctrl1 and gray rectangle represents Ctrl2. Data are mean ± SEM. One-way ANOVA followed by Tukey’s *post hoc* test was used for statistical analysis: ^∗^*P* < 0.05, §*P* < 0.001. PPA, peak-to-peak amplitude; PPD, peak-to-peak duration; PPS, peak-to-peak slope; CV, conduction velocity; SD, standard deviation.

Clinically, both BrS patients experience severe arrhythmias and reveal first-degree AV block in the ECGs. To investigate whether the arrhythmic predisposition is exhibited in the monolayer culture of iPSC-CMs, we applied multi-electrode array (MEA) to compare the conduction properties of spontaneous electrical excitations in 3-month-old Ctrl- and BrS-CMs (Figure 2). The differences between Ctrl- and BrS-CMs in the FP metrics PPD (ms), PPA (mV), and PPS (V/s) were distinct after enlargement (Figure 2A). The beating frequency of BrS1-CMs (0.46 ± 0.07 Hz, *n* = 23) and BrS2-CMs (0.44 ± 0.02 Hz, *n* = 19) was comparable with Ctrl-CMs (0.45 ± 0.03 Hz, *n* = 37, Figure 2C). However, BrS-CMs revealed a smaller PPA and PPS but a prolonged PPD when compared to Ctrl-CMs (Figures 2D–F). In Figure 2B, typical excitation patterns and local activation times in reference to the trigger point were shown for both Ctrl2- and BrS1-CMs. The excitation of Ctrl2-CMs spread from one corner (the lower left corner as the trigger point) of the MEA to the other in around 10 ms (Figure 2B) whereas BrS1-CMs needed around 45 ms for the same distance (Figure 2B), indicating pronounced conduction slowing in BrS-CMs when compared to Ctrl-CMs. Quantitative analysis revealed that the CV of Ctrl-CMs was 15.47 ± 1.05 cm/s (*n* = 37), whereas 4.7 ± 0.44 and 4.4 ± 0.49 cm/s were observed in BrS1-CMs (*n* = 23, *P* < 0.001) and BrS2-CMs (*n* = 19, *P* < 0.001), respectively (Figure 2G). This dramatic difference can be vividly observed in our recorded movies (Supplementary Videos 1, 2). Moreover, BrS-CMs exhibited increased inter-beat interval variability compared to Ctrl-CMs, as demonstrated by the SD of the inter-beat interval normalized to the mean of the inter-beat intervals (Figure 2H).

### Reduced Peak *I*_Na_ and Delayed Steady-State Activation of Sodium Channels in BrS-CMs

**FIGURE 3:**
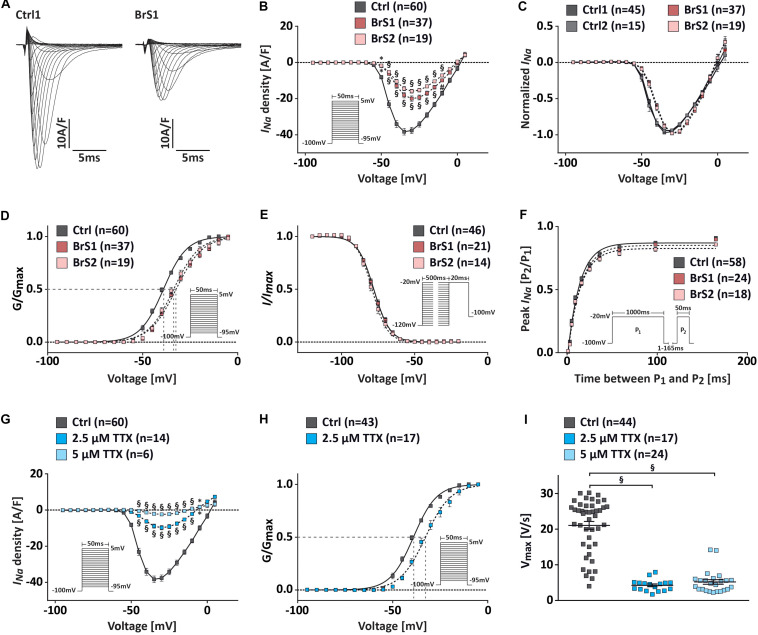
*I*_Na_ characterization in Ctrl- and BrS-CMs. **(A)** Examples of original *I*_Na_ traces elicited by 5 mV-step depolarization from −95 to 5 mV at a holding potential of −100 mV. Left, Na^+^ current in one Ctrl1-CM. Right, Na^+^ current in one BrS1-CM. **(B)** Average *I*–*V* relationships of the *I*_Na_ in Ctrl- and BrS-CMs. The protocol is shown as inset. **(C)** Current–voltage relationships normalized to the maximum peak *I*_Na_ show a positive shift of the peak *I*_Na_ in BrS1- and BrS2-CMs compared to Ctrl1- and Ctrl2-CMs. No differences were observed between two controls or two BrS. **(D)** Average of the steady-state voltage dependence of activation of the *I*_Na_ in Ctrl- and BrS-CMs. The protocol is shown as inset. **(E)** Average of the steady-state voltage dependence of inactivation of *I*_Na_ in Ctrl- and BrS-CMs. The protocol is shown as inset. **(F)** Average of the recovery from inactivation of *I*_Na_ in Ctrl- and BrS-CMs. The protocol is shown as inset. **(G)** Average current–voltage relationships of the *I*_Na_ in Ctrl-CMs with and without 2.5 or 5 μM TTX treatment. Protocol is shown as inset. **(H)** Average steady-state voltage dependence of activation in Ctrl-CMs treated with and without 2.5 μM TTX. Protocol is shown as inset. **(I)** *V*_max_ in Ctrl-CMs with and without 2.5 or 5 μM TTX treatment. Data are presented as mean ± SEM. Two-way repeated measures ANOVA **(B–H)** and one-way ANOVA followed by Tukey’s *post hoc* test were used for statistical analysis: ^∗^*P* < 0.05, #*P* < 0.01, §*P* < 0.001.

To assess the effect of the *SCN5A* mutation p.S1812X on the electrophysiological properties of BrS-CMs, *I*_Na_ density and gating properties of sodium channels were compared in 3-month-old BrS-CMs versus Ctrl-CMs using the whole-cell voltage-clamp technique (Figure 3). We observed no differences in the current density, voltage dependence, and gating properties of sodium channels between Ctrl1- and Ctrl2-CMs (Supplementary Figure 4). For a better overview and comparison of the data, we pooled the data of Ctrl1- and Ctrl2-CMs, as shown in Figure 3. Representative examples of *I*_Na_ recordings were shown in Figure 3A. Compared to the Ctrl-CMs, the *I*_Na_ densities were significantly reduced in BrS1- and BrS2-CMs (Figure 3B). The maximal peak *I*_Na_ in Ctrl-CMs (*n* = 60) at −35 mV showing −38.3 ± 1.8 A/F was reduced by more than 50% in BrS-CMs showing −18.1 ± 1.5 A/F in BrS1-CMs (*n* = 37, *P* < 0.001) and −16.7 ± 1.4 A/F in BrS2-CMs (*n* = 19, *P* < 0.001). There was no significant difference in the current–voltage relationship and the *I*_Na_ densities between BrS1- and BrS2-CMs (Figure 3B). However, current–voltage relationships normalized to the maximum peak *I*_Na_ showed a shift of peak current toward the positive direction in BrS-CMs compared to Ctrl-CMs (Figure 3C).

To study the changes in channel kinetics, we compared the steady-state (in)activation and the recovery from inactivation of the sodium channel in BrS-CMs versus Ctrl-CMs. The steady-state activation curve was significantly shifted in a depolarizing direction in BrS-CMs compared to Ctrl-CMs (Figure 3D). *V*_1__/__2_ was −39.1 ± 0.2 mV for Ctrl-CMs (*n* = 60), −32.4 ± 0.2 mV for BrS1-CMs (*n* = 37, *P* < 0.001), and −33.7 ± 0.3 mV for BrS2-CMs (*n* = 25, *P* < 0.001). Slope factor *k*_∞_ was 6.1 ± 0.2 mV for Ctrl-CMs, significantly smaller than 6.9 ± 0.2 mV for BrS1-CMs (*P* < 0.001) and 6.7 ± 0.2 mV for BrS2-CMs (*P* < 0.05) (Supplementary Table 1). The steady-state inactivation of sodium channels in BrS-CMs did not differ significantly from Ctrl-CMs (Figure 3E and Supplementary Table 1). Next, we determined the fraction (*A*) of sodium channels recovered from an inactivation pulse (1000 ms at −20 mV) and the time constant (*τ_rec_*) for this recovery. We found a slight but not significant slowing of the recovery from the inactivation in BrS-CMs versus Ctrl-CMs (Figure 3F and Supplementary Table 1). However, no difference in persistent *I*_Na_ was detected in our study (Supplementary Figures 4F,G).

To assess whether the reduced *I*_Na_ density in BrS-CMs is related to the positive shift of the steady-state activation, we treated Ctrl-CMs with the Na^+^ channel blocker tetrodotoxin (TTX; 2.5 and 5 μM, Figures 3G–I). The treatment with 5 μM TTX resulted in a robustly reduced *I*_Na_, decreasing maximal *I*_Na_ to 5% of the basal level (Figure 3G). The peak *I*_Na_ density at −35 mV in Ctrl-CMs treated with 2.5 μM TTX (−9.7 ± 1.2 A/F, *n* = 14) showed a significant reduction compared to non-treated Ctrl-CMs (−38.3 A/F, *n* = 60) (Figure 3G). We also observed a significant positive shift in the steady-state activation, as demonstrated by the change in *V*_1__/__2_ in Ctrl-CMs treated with 2.5 μM TTX (−32.7 ± 0.3 mV, *n* = 14) compared to non-treated Ctrl-CMs (−39.1 ± 0.2 mV, *n* = 60, *P* < 0.001) (Figure 3H). These data suggest that the reduced *I*_Na_ density in BrS-CMs is associated with the positive shift of the steady-state activation.

To study whether the blockage of Na^+^ channel by TTX can mimic the FP properties observed in BrS-CMs, we treated Ctrl-CMs with 2.5 μM TTX for 10 min. We found that the application of TTX in Ctrl-CMs led to a significantly reduced PPA, PPS, and CV, but a prolonged PPD (Supplementary Figure 5). In addition, TTX reduced beating frequency (Supplementary Figure 5). Taken together, these data indicate that BrS-CMs recapitulate the conduction abnormalities seen in patients with loss-of-function sodium channel mutations, and the conduction slowing can be evoked in Ctrl-CMs by TTX and is associated with the diminution of *I*_Na_.

### Augmented *I*_to_ Current and Altered Window Current of *I*_CaL_ in BrS-CMs

**FIGURE 4:**
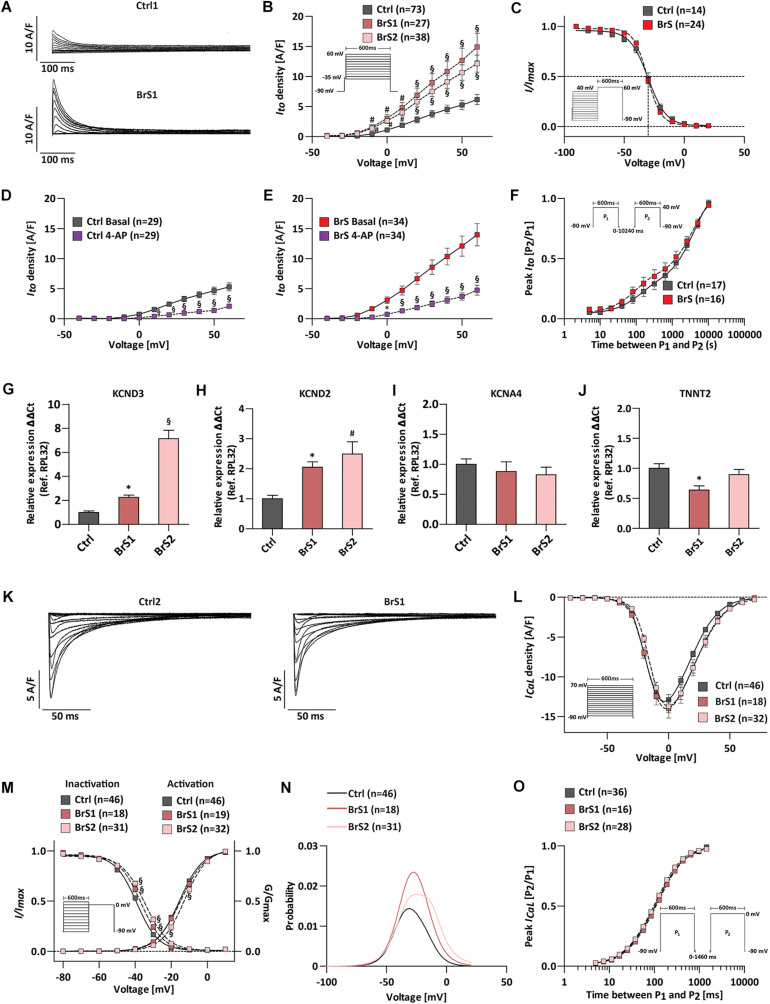
*I*_CaL_ and *I*_to_ characterization of Ctrl- and BrS-CMs. **(A)** Examples of original *I*_to_ traces elicited by 10 mV-step depolarization from −40 to 60 mV at a holding potential of −90 mV. Top, *I*_to_ in one Ctrl1-CM. Bottom, *I*_to_ in one BrS1-CM. **(B)** Average *I*–*V* relationships of the *I*_to_ current in Ctrl- and BrS-CMs. Protocol is shown as inset. **(C)** Average of the steady-state voltage dependence of inactivation *I*_to_ current in Ctrl- and BrS-CMs. The pulse protocol is shown as inset. No significant differences were observed. The *I*_to_ current density in Ctrl-CMs **(D)** and BrS-CMs **(E)** with and without 4-AP (1 mM) treatment for 1 min. **(F)** Average of recovery from inactivation of *I*_to_ current in Ctrl- and BrS-CMs. The pulse protocol is shown as inset. No significant differences were observed. Expression profile of *I*_to_-related genes: *KCND3* **(G)**, *KCND2* **(H)**, *KCNA4* **(I)**, and *TNNT2* **(J)**. Data were presented relative to *RPL32* expression (Ctrl: *n* = 6, BrS1: *n* = 6, BrS2: *n* = 6 independent differentiation experiments). **(K)** Examples of original *I*_CaL_ traces of a Ctrl2-CM and a BrS1-CM. **(L)** Average *I*–*V* relationships of the *I*_CaL_ in Ctrl-, BrS1-, and BrS2-CMs. The protocol is shown as inset. **(M)** The steady-state of activation (*G*/*G*_max_) and steady-state of inactivation (*I*/*I*_max_) are overlaid to show the window current of *I*_CaL_. Window currents are the areas under the intersecting current–voltage curves. The impulse for inactivation is shown as inset. **(N)** The probability being within *I*_CaL_ window current is plotted. **(O)** Average of recovery from inactivation of *I*_CaL_ in Ctrl-, BrS1-, and BrS2-CMs. The pulse protocol is shown as inset. Data are presented as mean ± SEM. Two-way repeated measures ANOVA was used for *I*_to_ and *I*_CaL_ statistical analysis. One-way ANOVA was used for gene expression analyses. ^∗^*P* < 0.05, #*P* < 0.01, §*P* < 0.001.

Previous studies reported that in the presence of weak *I*_Na_, the unopposed outward K^+^ current *I*_to_ cause accentuation of the AP notch in the right ventricular epicardium, resulting in accentuated J wave and ST-segment elevation associated with the Brugada pattern (Szel and Antzelevitch, 2014). To test whether *I*_to_ is pivotal in generating the disease phenotype in BrS, we recorded *I*_to_ from single iPSC-CMs (Figure 4). The representative *I*_to_ traces of Ctrl1-CMs and BrS1-CMs were shown in Figure 4A. Surprisingly, the BrS1-CMs and BrS2-CMs exhibited significantly larger *I*_to_ in comparison to Ctrl-CMs (Figure 4B). The maximum peak currents at +60 mV in BrS1-CMs (14.91 ± 2.27 A/F, *n* = 27, *P* < 0.001) and BrS2-CMs (12.14 ± 1.42 A/F, *n* = 38, *P* < 0.001) were 2.4 and 1.9 times bigger than those in Ctrl-CMs (6.14 ± 0.85 A/F, *n* = 73) (Figure 4B). When the cells were treated with 4-aminopyridine (4-AP), an *I*_to_ inhibitor, 60.9% reduction of the currents was observed in Ctrl-CMs (from 5.29 ± 0.67 to 2.07 ± 0.24 A/F, paired, *n* = 29; Figure 4D), which is comparable to 65.9% reduction in BrS-CMs (from 13.97 ± 1.8 to 4.8 ± 0.84 A/F, paired, *n* = 34; Figure 4E), indicating the measured currents are the 4-AP-sensitive *I*_to_. Nevertheless, we did not detect any significant differences in *V*_1__/__2_ for the steady-state inactivation between Ctrl-CMs (−30.41 ± 1.6 mV, *n* = 14) and BrS-CMs (−30.42 ± 0.72 mV, *n* = 24) (Figure 4C and Supplementary Table 1). Recovery from the inactivation of *I*_to_ in Ctrl-CMs showed fast (fast *τ_rec_*, 279.7 ± 121.1 ms) and slow phases (slow *τ_rec_*, 2854 ± 581.7 ms), which were comparable to the fast (fast *τ_rec_*, 211.9 ± 135.8 ms) and slow phases (slow *τ_rec_*, 2773 ± 462.6 ms) in BrS-CMs, respectively (Figure 4F and Supplementary Table 1). Using real-time PCR technique, we assessed the expression of *I*_to_-related genes *KCND2/3* and *KCNA4*. It turned out the expressions of *KCND2* and *KCND3* were significantly higher in BrS1- and BrS2-CMs when compared to Ctrl-CMs (Figures 4G,H). However, no difference regarding the expression of *KCNA4* was found between Ctrl- and BrS-CMs (Figure 4I). The *TNNT2* expression was normalized to reference gene *RPL32* like the other three genes (Figure 4J).

We further checked the *I*_CaL_ and did not detect significant differences in the peak *I*_CaL_ at 0 mV in BrS1-CMs (−13.99 ± 1.18 A/F, *n* = 18) or BrS2-CMs (−13.71 ± 0.8 A/F, *n* = 32) compared to Ctrl-CMs (−12.88 ± 0.66 A/F, *n* = 46) (Figures 4K,L). The curve of the steady-state activation was shifted to more depolarizing voltage in BrS2-CMs (*V*_1__/__2_ = −12.51 ± 0.50 mV, *n* = 32, *P* < 0.001) but not in BrS1-CMs (*V*_1__/__2_ = −14.45 ± 0.84 mV, *n* = 19, *P* > 0.05) when compared to Ctrl-CMs (*V*_1__/__2_ = −15.54 ± 0.38 mV, *n* = 46) (Figure 4M and Supplementary Table 1). The steady-state inactivation curves were also significantly shifted toward more depolarizing voltage for BrS1-CMs (*V*_1__/__2_ = −36.83 ± 0.38 mV, *n* = 18, *P* < 0.001) and BrS2-CMs (*V*_1__/__2_ = −34.61 ± 0.40 mV, *n* = 31, *P* < 0.001) when compared to Ctrl-CMs (*V*_1__/__2_ = −39.18 ± 0.22 mV, *n* = 46) (Figure 4M and Supplementary Table 1). Notably, the shifts in the voltages for half-(in)activation *V_1__/__2_* and a slight increase in slopes of (in)activation *k*_∞_ resulted in the significant increases in the window current (area under the curves in Figure 4N). After calculating with a window current probability formula, we found the probabilities of the *I*_CaL_ window current increased 1.59 times for BrS1-CMs and 1.56 times for BrS2-CMs when compared to Ctrl-CMs (Figure 4N). As window currents are steady-state currents, bigger window currents indicate the increased Ca^2+^ influx that may contribute to the formation of afterdepolarization and arrhythmia in BrS-CMs. The maximum ratio of peak *I*_CaL_ [*P*_2_/*P*_1_] of the recovery from the inactivation for Ctrl-CMs is comparable to those for BrS1-CMs and BrS2-CMs (Figure 4O and Supplementary Table 1). Taken together, our results demonstrate that BrS-CMs exhibit distinct anomalies not only in Na^+^ channel function but also in K^+^ and Ca^2+^ channel functions compared to Ctrl-CMs, which may result in the abnormal AP phenotype.

### Reduced *V*_max_ and Increased Irregular Repolarization in APs of BrS-CMs

**FIGURE 5:**
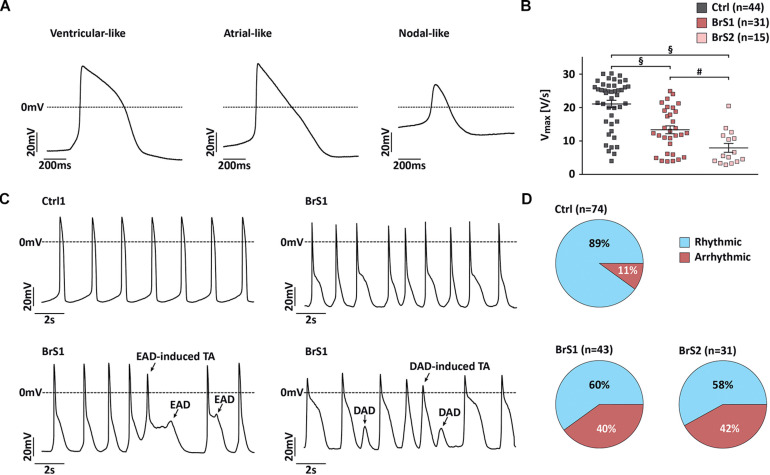
Action potential characterization of Ctrl- and BrS-CMs. **(A)** Representative traces of spontaneous action potentials (APs) measured in ventricular-, atrial-, and nodal-like CMs. **(B)** *V*_max_ in ventricular- and atrial-like BrS- and Ctrl-CMs. Data are presented as mean ± SEM. One-way ANOVA followed by Tukey’s *post hoc* test was used for statistical analysis: #*P* < 0.01, §*P* < 0.001. **(C)** Representative traces of spontaneous AP recordings from Ctrl- and BrS-CMs. **(D)** Percentage of Ctrl- and BrS-CMs with spontaneously (ar)rhythmic beating. Two-tailed Fisher’s exact test was used for statistical analysis.

According to the following stringent criteria, the majority of 3-month-old iPSC-CMs (81%) reveal ventricular-like APs by using the C-clamp mode of patch-clamp technique (Figure 5 and Supplementary Table 2). Ventricular-like APs exhibit a relatively negative resting membrane potential (RMP; <−60 mV), a rapid maximal upstroke velocity (*V*_max_), a prominent plateau phase, and an AP amplitude (APA) over 95 mV. Atrial-like APs reveal similar properties as ventricular-like APs but lack the plateau phase. Nodal-like APs exhibit a more positive RMP (≥−55 mV), a slow *V*_max_ (≤5 V/s), and an APA < 85 mV. Overall, no significant differences in RMP and APA were observed in BrS-CMs compared to Ctrl-CMs (Supplementary Table 2). Since sodium channels are responsible for the fast upstroke of the atrial and ventricular APs, as expected, the *V*_max_ was significantly slower in spontaneously beating ventricular- and atrial-like BrS1- and BrS2-CMs compared to Ctrl-CMs (Figure 5A). Similarly, Ctrl-CMs after the treatment with TTX showed a slower *V*_max_ (Figure 3I). We also observed a significantly slower *V*_max_ in BrS2-CMs than in BrS1-CMs (Figure 5B), which is consistent with the more pronounced *I*_Na_ reduction in BrS2-CMs than in BrS1-CMs. Additionally, we found a pronounced AP notch in BrS-CMs compared to Ctrl-CMs (Figure 5C), which is in line with the augmented *I*_to_ density in BrS-CMs. Notably, we detected prominent irregularities in spontaneous AP recordings in BrS-CMs, including early afterdepolarization (EAD), EAD-triggered activities, delayed afterdepolarization (DAD), and DAD-triggered activities (Figure 5C). We observed the irregularities in 40% of BrS1-CMs (*n* = 43; *P* < 0.001, Fisher’s exact test) and 42% of BrS2-CMs (*n* = 31; *P* < 0.001) compared to 11% of Ctrl-CMs (*n* = 74, Figure 5D). Taken together, these results demonstrate that BrS-CMs exhibit distinctly abnormal AP phenotype compared with Ctrl-CMs.

### Assessment of Potential Pharmacotherapies Using BrS-CMs

**FIGURE 6:**
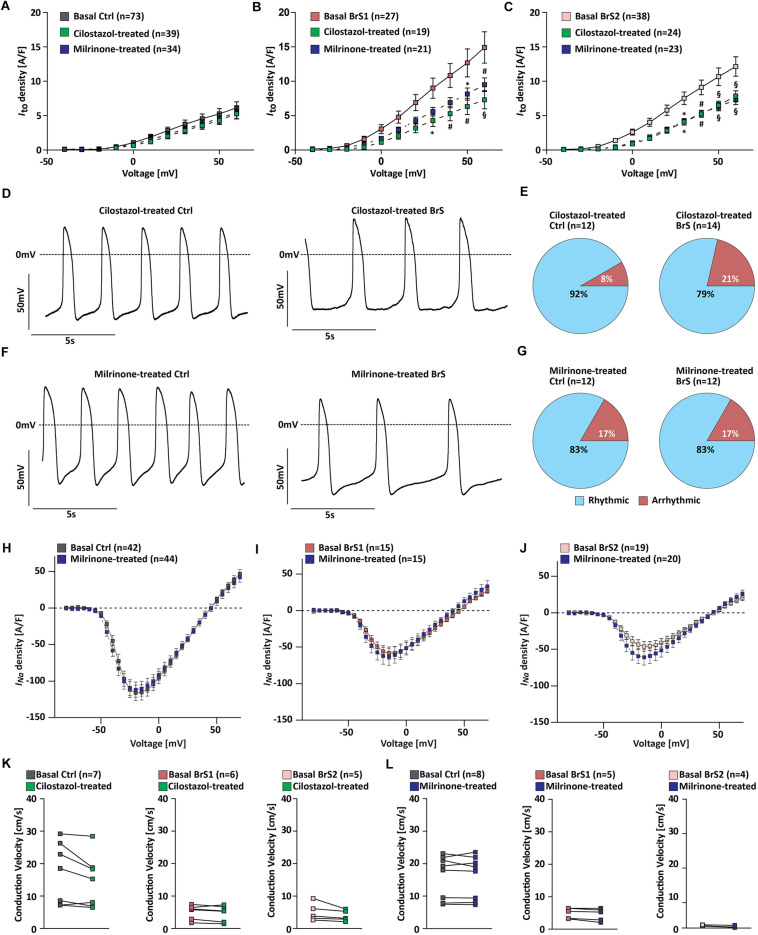
Effects of PDE inhibitor cilostazol or milrinone on electrophysiological properties of Ctrl- and BrS-CMs. Average current–voltage relationships of *I*_to_ in Ctrl-CMs **(A)**, BrS1-CMs **(B)**, and BrS2-CMs **(C)** with and without cilostazol (10 μM) or milrinone (2.5 μM) treatment for half an hour. Data are presented mean ± SEM. **(D)** Representative original action potential (AP) traces of Ctrl- and BrS-CMs after 10 μM cilostazol treatment. **(E)** Percentages of Ctrl- and BrS-CMs with rhythmic and arrhythmic APs after 10 μM cilostazol treatment. **(F)** Representative original AP traces of Ctrl- and BrS-CMs after 2.5 μM milrinone treatment. **(G)** Percentages of Ctrl- and BrS-CMs with rhythmic and arrhythmic APs after 2.5 μM milrinone treatment. Average *I*–*V* curves of *I*_Na_ (automated patch-clamp) in Ctrl-CMs **(H)**, BrS1-CMs **(I)**, and BrS2-CMs **(J)** with and without 2.5 μM milrinone treatment for half an hour. No statistically significant differences were observed. **(K)** Quantitative analysis of the conduction velocity in Ctrl-, BrS1-, and BrS2-CMs with and without cilostazol (10 μM) treatment. No statistically significant differences were observed. **(L)** Quantitative analysis of the conduction velocity in Ctrl-, BrS1, and BrS2-CMs with and without 2.5 μM milrinone treatment. No statistically significant differences were observed. Two-way repeated measures ANOVA **(A–C,H–J)** and two-tailed paired Student’s *t*-test **(K,L)** were used for statistical analysis: ^∗^*P* < 0.05, #*P* < 0.01, §*P* < 0.001.

Given that two clinically used PDE inhibitors cilostazol and milrinone have been shown to suppress the hypothermia-induced ventricular tachycardia/ventricular fibrillation by reversing the repolarization abnormalities (Gurabi et al., 2014), we tested their effects on our iPSC-CMs at therapeutically relevant concentrations (Szel et al., 2013). After 10 μM cilostazol treatment of BrS-CMs for half an hour, we observed a 50.9% reduction of *I*_to_ in BrS1-CMs (from 14.91 to 7.3 A/F) and a 35.4% reduction in BrS2-CMs (from 12.14 to 7.8 A/F) at +60 mV pulse stimulation, whereas only a slight but not significant *I*_to_ reduction of 14.4% was observed for the Ctrl-CMs (from 6.14 to 5.25 A/F) (Figures 6A–C). The similar pattern was found when we tested the treatment with 2.5 μM milrinone at the same condition: *I*_to_ was significantly reduced from 14.91 to 9.5 A/F in BrS1-CMs (36.3% reduction) and from 12.14 to 7.3 A/F in BrS2-CMs (40% reduction), but only a 10.9% reduction in Ctrl-CMs (from 6.14 to 5.7 A/F) (Figures 6A–C).

Moreover, we assessed whether these two drugs can alleviate the arrhythmic beating degree of BrS-CMs. We found that the treatment of BrS-CMs with cilostazol led to the decrease of cells with arrhythmic beating from 40.5% (*n* = 74) to 21% (*n* = 14) (Figures 6D,E). We detected the similar effect of milrinone on BrS-CMs and the arrhythmic beating degree of BrS-CMs dropped to 17% (*n* = 12) (Figures 6F,G). Both cilostazol and milrinone treatment had no obvious effect on Ctrl-CMs (Figures 6D–G). The most interesting thing is that no EAD- or EAD-triggered activities were observed in these AP recordings.

We further studied the effect of milrinone on *I*_Na_ using the automated patch-clamp system and found no significant changes in *I*_Na_ in both Ctrl- and BrS-CMs after 2.5 μM milrinone treatment for half an hour (Figures 6H–J). In line with these data, there were no significant effects of cilostazol and milrinone on the CV in Ctrl-, BrS1-, and BrS2-CMs (Figures 6K,L), but inter-beat interval variability was slightly reduced (Supplementary Figures 4H–K).

## Discussion

In this study, we applied patient-specific iPSC-CMs carrying the non-sense mutation p.S1812X in *SCN5A* and investigated the pathophysiological phenotypes and possible pharmacological therapy. Our results demonstrate the ability of patient-specific BrS-CMs to recapitulate the loss-of-function of Na_V_1.5 resulting from the truncation of SCN5A as evidenced by the significant reduction of peak *I*_Na_, the significantly slower of the steady-state activation and the 69% reduction of Na_V_1.5 α-subunit protein expression. In line with these findings, we also observed the decreased AP upstroke velocity and the conduction slowing in BrS-CMs. Notably, we also found the increased *I*_to_ and the augmented window current of *I_Ca__L_* in BrS-CMs when compared to Ctrl-CMs. We believe that the impaired interplay between *I*_Na_, *I*_to_, and *I*_CaL_ may be involved in the generation of arrhythmia in BrS-CMs. Furthermore, we showed the positive effect of PDE inhibitors cilostazol and milrinone on the reduction of the arrhythmia in BrS-CMs.

In this study, we, for the first time, applied the MEA technology and demonstrated the conduction slowing in BrS-CMs. It is well known that the *I*_Na_ and the electrically coupling degree of gap junctions are the key determinants of cardiac conduction (Kucera et al., 2002). We demonstrated that the *I*_Na_ density was reduced in BrS-CMs, which is consistent with other observations in iPSC-CMs with BrS-related mutations in *SCN5A* (Liang et al., 2016; Ma et al., 2018). Sodium channels play an important role in phase 0 of the cardiac AP and determine the upstroke velocity (Satin et al., 2004). In BrS-CMs, *V*_max_ was significantly lower compared to Ctrl-CMs, consistent with observed reduction in peak *I*_Na_ density. These findings were also demonstrated in patient-specific iPSC-CMs harboring the *SCN5A*^1795insD^ mutation (Davis et al., 2012). Our findings that the *I*_Na_ activation curve shifted toward more positive potential with an increased slope factor are in accordance with the later activation of sodium channels resulted from loss-of-function mutations in *SCN5A* as previously reported for BrS patients (Wilde and Amin, 2018). Additionally, the diminution of *I*_Na_ and the altered gating properties are clearly associated with the expression reduction of full-length Na_V_1.5 protein and the disrupted localization at the cell surface due to the non-sense mutation p.S1812X in *SCN5A* in BrS-CMs. Our data are in line with the previous study demonstrating that in HEK293 cells overexpressing the wild-type and the BrS-associated mutant p.V2016M *SCN5A* together, the surface expression of Na_V_1.5 is reduced due to its disturbed interaction with SAP97 via a PDZ domain (the last three residues SIV of Na_V_1.5), with a subsequent decrease of *I*_Na_ (Shy et al., 2014).

In line with previous studies (Kucera et al., 2002; Edokobi and Isom, 2018), we found the co-localization of Na_V_1.5 and Cx43 at the sites of cell-to-cell appositions in both Ctrl- and BrS-CMs, however, in BrS-CMs the co-localization of Na_V_1.5 and Cx43 seems altered. Physical and functional interactions of sodium channel complex with gap junction and desmosomal components at intercalated discs in CMs are essential for cardiac excitability and conduction (Delmar, 2012; Cerrone and Delmar, 2014). Previous study demonstrated that loss of the gap junction protein Cx43 resulted in diminished Na_V_1.5 expression at intercalated discs (Jansen et al., 2012). In our study, we observed a reduced expression of Cx43 in BrS-CMs, this brings up an open question: how the reduced expression of Na_V_1.5 regulates the Cx43 expression, which should be clarified in future studies. Nonetheless, our data indicate that BrS-CMs with loss-of-function mutation p.S1812X in *SCN5A* reveal some pathophysiological phenotypes including the conduction slowing, supporting the “depolarization disorder” theory as the underlying mechanism of BrS.

Notably, we show that *I*_to_ is present in 3-month-old iPSC-CMs and, to our surprise, the *I*_to_ was significantly larger in BrS-CMs than in Ctrl-CMs. However, genetic analysis in both patients did not find mutations in *KCND3*, *KCNQ1*, *HERG*, and *KCNE1* (Schulze-Bahr et al., 2003). The 4-AP sensitivity experiments confirmed that the currents we recorded were *I*_to_. Moreover, we observed a prominent AP notch in BrS-CMs. Previous studies showed that *I*_to_ is pivotal in generating the disease phenotype in BrS (Di Diego et al., 2002; Giudicessi et al., 2011). The direct relationship between *I*_to_ and BrS was first reported by applying the *I*_to_ activator NS5806 in isolated canine ventricular wedge preparations. NS5806 could increase phase 1 and notch amplitude of the AP in the epicardium, but not in the endocardium, and accentuate J-wave in the ECG (Calloe et al., 2009). It is well accepted that the orderly sequence of repolarization in the heart is linked to heterogeneous distribution of the fast component of *I*_to_ in right ventricle epicardium and endocardium. The highest density of *I*_to_ is seen in epicardial CMs, whereas the lowest density is observed in endocardial CMs (Litovsky and Antzelevitch, 1988; Wettwer et al., 1994; Costantini et al., 2005). The changes in repolarization might lead to the so-called phase 2 re-entry and polymorphic ventricular tachycardia. One limitation of our study is that the *in vitro* BrS-CM model cannot recapitulate the *in vivo* heterogeneous distribution of *I*_to_ in right ventricular epicardium and endocardium. Nonetheless, our data demonstrate that the increased *I*_to_ in BrS-CMs is associated to the disease phenotype, and are in line with the previous studies (Delpon et al., 2008; Giudicessi et al., 2011; Perrin et al., 2014). Gain-of-function mutations in *KCND3* encoding K_V_4.3 were found in patients with BrS, leading to the loss of the AP dome in the modified Luo-Rudy II AP model as a result of the increased *I*_to_ (Giudicessi et al., 2011). A mutation in *KCNE3* encoding a β-subunit of the K_V_4.3 channel was found in a patient with BrS, which resulted in the reduction of its inhibitory effect on the K_V_4.3 channel, leading to an increase in *I*_to_ (Delpon et al., 2008). Additionally, the mutation p.D612N in *KCND2* coding for K_V_4.2 was identified responsible for the increased J-wave manifestation (Perrin et al., 2014). On the contrary, two recent studies reported some different findings. Different with our increased mRNA expression of *KCND2/3*, a significant decrease in the gene expression of *KCND3* was revealed in CMs derived from iPSCs generated from two BrS patients carrying the *SCN5A* R620H-R811H mutation and *SCN5A* Δ1397 mutation, respectively (Liang et al., 2016). Moreover, the *I*_to_ current density remained unaltered in BrS iPSC-CMs with *SCN5A* A226V-R1629X variant (Ma et al., 2018). Although our data showing the increased *I*_to_ in BrS-CMs support the “repolarization disorder” theory of BrS, further studies need to investigate how the *SCN5A* mutation p.S1812X affects the transient-outward potassium channels, especially when we did not observe changes in K_V_4.3 protein expression in BrS-CMs. We postulate that the augmented *I*_to_ may result from the post-translational modification of Kv4.3 channel and impaired protein-protein interaction. It is worth mentioning that there is an inverse relationship between the maximum velocities of depolarization and repolarization, as demonstrated in murine isolated ventricular CMs as well as in HEK293 cells overexpressing both K_V_4.3 and Na_V_1.5 (Portero et al., 2018). Therefore, it is of vital importance to further study the entire molecular complexity, in which Na_V_1.5 is naturally embedded in human CMs and to assess the functional interactions of different ion channels.

Moreover, in our study higher arrhythmic tendencies in BrS-CMs were observed. Spontaneous AP recordings revealed a higher percentage of BrS-CMs with arrhythmogenic events, and FP recordings displayed the increased SD of the inter-beat intervals. Unlike type 3 long QT syndrome, in which most of the *SCN5A* mutations cause a significantly enhanced persistent *I*_Na_ contributing to the life-threatening ventricular arrhythmias (Malan et al., 2011; Ma et al., 2013), the mutation p.S1812X in our study did not alter the persistent *I*_Na_ in BrS-CMs. To our surprise, the window current probability of *I_Ca__L_* was significantly increased in BrS-CMs compared to Ctrl-CMs, which is associated with the alterations in the steady-state activation and steady-state inactivation of the L-type calcium channels. Previous studies demonstrated that the increased *I*_CaL_ window current contributed to the EAD formation and EAD-mediated arrhythmias (Madhvani et al., 2015). In iPSC-CMs derived from BrS patients without identified mutations, no significant changes in the steady-state activation and steady-state inactivation of the L-type calcium channels were observed, but the probability of *I_Ca__L_* window current was not calculated (Veerman et al., 2016). Future studies should investigate how the different channels in CMs, including the channels responsible for *I*_CaL_, *I*_to_, and *I*_Na_, co-operate and regulate the arrhythmia in BrS-CMs.

Based on the *I*_to_ augment in our BrS-CMs, we speculate that the alleviation of AP arrhythmia could be possibly achieved through the reduction of *I*_to_ currents. Cilostazol and milrinone are two oral PDE3 inhibitors, which might be potentially antiarrhythmic drugs for BrS patients (Tsuchiya et al., 2002). Previous study reported that the ventricular fibrillation of a 67-year-old patient with BrS was prevented by oral cilostazol administration (Tsuchiya et al., 2002). In the setting of an arterially perfused right ventricular wedge, cilostazol and milrinone could suppress arrhythmogenesis associated with BrS (Szel et al., 2013; Szel and Antzelevitch, 2014). The effects of cilostazol and milrinone may be related to an elevation of intracellular cyclic AMP concentration via inhibition of PDE activity, and consequently to suppression of *I*_to_ and to an increase in *I*_Ca_, as previously discussed (Tsuchiya et al., 2002; Szel et al., 2013; Szel and Antzelevitch, 2014). Patocskai et al. (2016) demonstrated that both cilostazol and milrinone reduced *I*_to_ in canine single left ventricular CMs, and addition of cilostazol or milrinone to the coronary perfusate restored the AP dome at all epicardial sites, reduced epicardial and transmural dispersion of repolarization, decreased J point and ST segment elevation, and terminated all arrhythmic activity in an experimental model of early repolarization syndrome. In our study, inhibition effects of cilostazol and milrinone on *I*_to_ and arrhythmogenic events in BrS-CMs suggest a new therapeutic potential to alleviate BrS probably via *I*_to_ reduction. Nevertheless, cilostazol and milrinone do not have effects on *I*_Na_ or CV.

## Conclusion

By using iPSC-CMs from two BrS patients harboring the *SCN5A* mutation p.S1812X, we demonstrate here that the mutation results in the reduced *I*_Na_ but augmented *I*_to_ and increased *I*_CaL_ window current probability as well as conduction slowing, indicating that both repolarization and depolarization disorders coexist in BrS-CMs. Our findings indicate that the electrophysiological mechanisms underlying conduction slowing and arrhythmia in BrS-CMs involve not only the Na_V_1.5 loss-of-function but also an impaired coordination of *I*_Na_, *I*_to_, and *I*_CaL_. Moreover, pharmacological treatments with the PDE3 inhibitors cilostazol and milrinone reduce the *I*_to_ and proarrhythmic events in BrS, suggesting their therapeutic potential for BrS patients.

## Data Availability Statement

All datasets presented in this study are included in the article/Supplementary Material.

## Ethics Statement

The studies involving human participants were reviewed and approved by the Institutional Ethics Committee of University Medical Center Göttingen (approval number 21/1/11) and of Technical University of Dresden (approval number EK 422092019) and carried out in accordance with the approved guidelines. The patients/participants provided their written informed consent to participate in this study.

## Author Contributions

WL, MiS, SW, and KG conceived the study and designed experiments. WL, MiS, XJL, MV, CSM, MaS, SC, GW, and S-MH performed experiments and acquired data. WL, MiS, XJL, SW, MV, MaS, and KG analyzed the data. WL, MiS, SW, LC, GH, LSM, AE-A, and KG contributed to the interpretation of the data. WL, MiS, and KG wrote the manuscript. All authors contributed to the article and approved the submitted version.

## Conflict of Interest

The authors declare that the research was conducted in the absence of any commercial or financial relationships that could be construed as a potential conflict of interest.
