# Supplementary material for: Disease Phenotypes and Mechanisms of iPSC-Derived Cardiomyocytes From Brugada Syndrome Patients With a Loss-of-Function SCN5A Mutation
Source: Front Cell Dev Biol. 2020 Oct 22;8:592893. doi: 10.3389/fcell.2020.592893 (PMC7642519; doi:10.3389/fcell.2020.592893)
Supplement: Supplementary file 3 [file Data_Sheet_1.pdf]

Disease phenotypes and mechanisms of iPSC-derived cardiomyocytes from Brugada syndrome patients with a loss-of-function SCN5A mutation

Wener Li[1#], Michael Stauske[2,3#], Xiaojing Luo[1], Stefan Wagner[2,6], Meike Vollrath[1], Carola S. Mehnert[1], Mario Schubert[1], Lukas Cyganek[2,3], Simin Chen[3], Sayed-Mohammad Hasheminasab[5,6], Gerald Wulf[4], Ali El-Armouche[1], Lars S. Maier[2,7], Gerd Hasenfuss[2,3], Kaomei Guan[1,2,3*]

[1]Institute of Pharmacology and Toxicology, Technische Universität Dresden, Fetscherstr. 74, 01307 Dresden, Germany; [2]Department of Cardiology and Pneumology, University Medical Center Göttingen, Robert-Koch-Str. 40, 37075 Göttingen, Germany; [3]German Center for Cardiovascular Research (DZHK), partner site Göttingen, Robert-Koch-Str. 40, 37075 Göttingen, Germany; [4]Department of Hematology and Oncology, University Medical Center Göttingen, Robert-Koch-Str. 40, 37075 Göttingen, Germany; [5]Department of Dermatology, Venereology and Allergy, Charité-Universitätsmedizin Berlin; [6]CCU Translational Radiation Oncology, German Cancer Consortium Core-Center Heidelberg, National Center for Tumor Diseases, Heidelberg University Hospital (UKHD) and German Cancer Research Center (DKFZ), Heidelberg Germany; [7]Clinic for Internal Medicine II, University Hospital Regensburg, Franz-Josef-Strauß-Allee 11, 93053 Regensburg, Germany

[#]These authors contributed equally to this work.

Supplementary Materials

Fig. S1

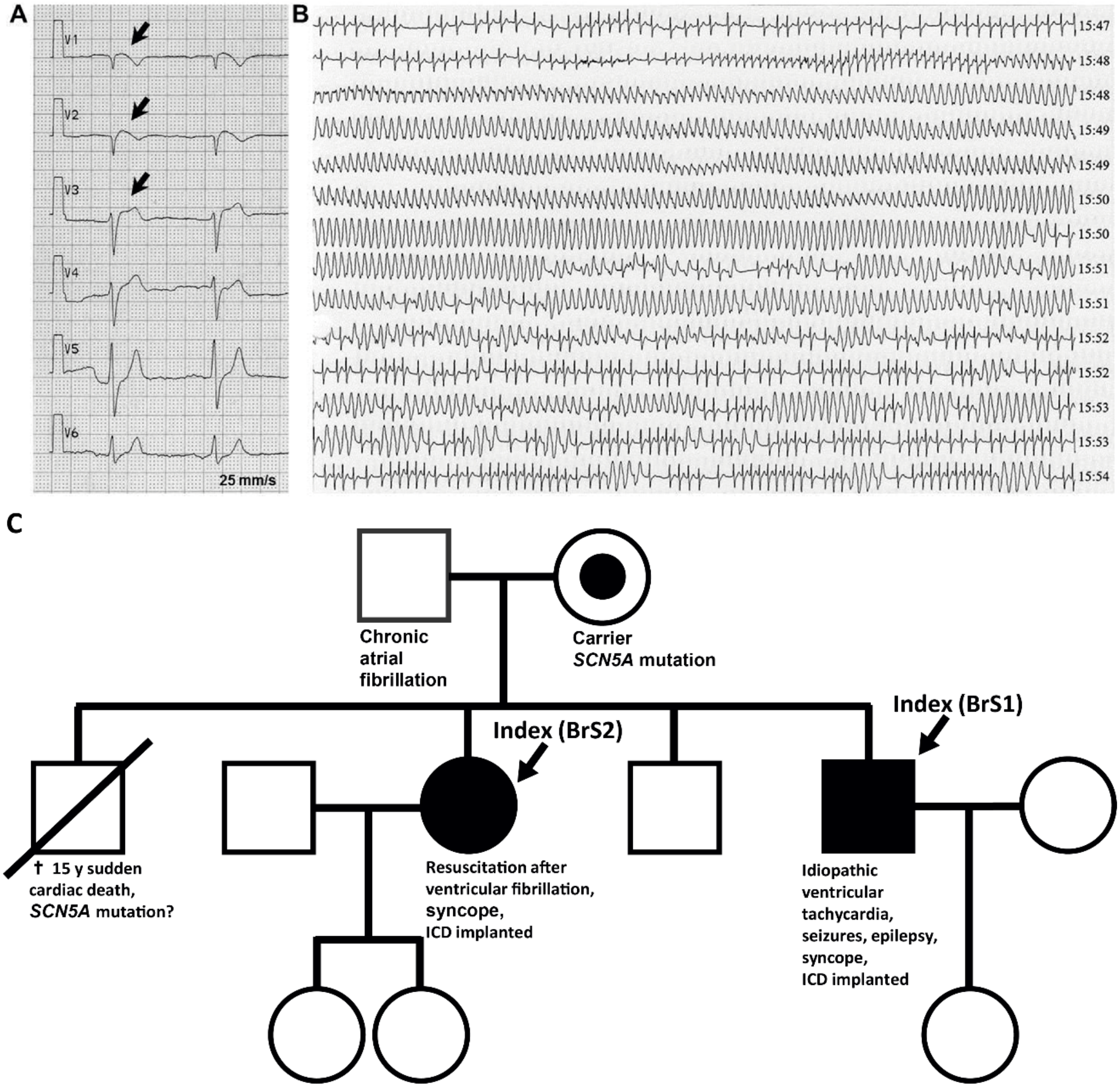

Supplementary Figure 1. Excerpts of 12-lead ECG from the BrS1 patient, and pedigree of his family. (**A**) Recordings from precordial leads V_1 – V_6 are shown. The recordings of the BrS1 patient show a slight coved-type ST-segment elevation in V_1 and V_2 (BrS type I ECG) and a more saddleback pattern in V_3 (BrS type II ECG), followed by a negative T-wave in each case. (**B**) Spontaneous ventricular tachycardia during recordings with Holter-ECG. (**C**) The male BrS patient (BrS1) suffers from idiopathic ventricular tachycardia, seizures and syncope. His sister (BrS2) had to be resuscitated after a collapse due to ventricular fibrillation. Both patients carry an implantable cardioverter-defibrillator (ICD). They are heterozygous for the point mutation c.C5435A in the *SCN5A* gene, which leads to a premature termination codon in exon 28 (p.S1812X). The mother is a carrier of the mutation. One brother died from sudden cardiac death at the age of 15. His genotype is unknown.

Fig. S2

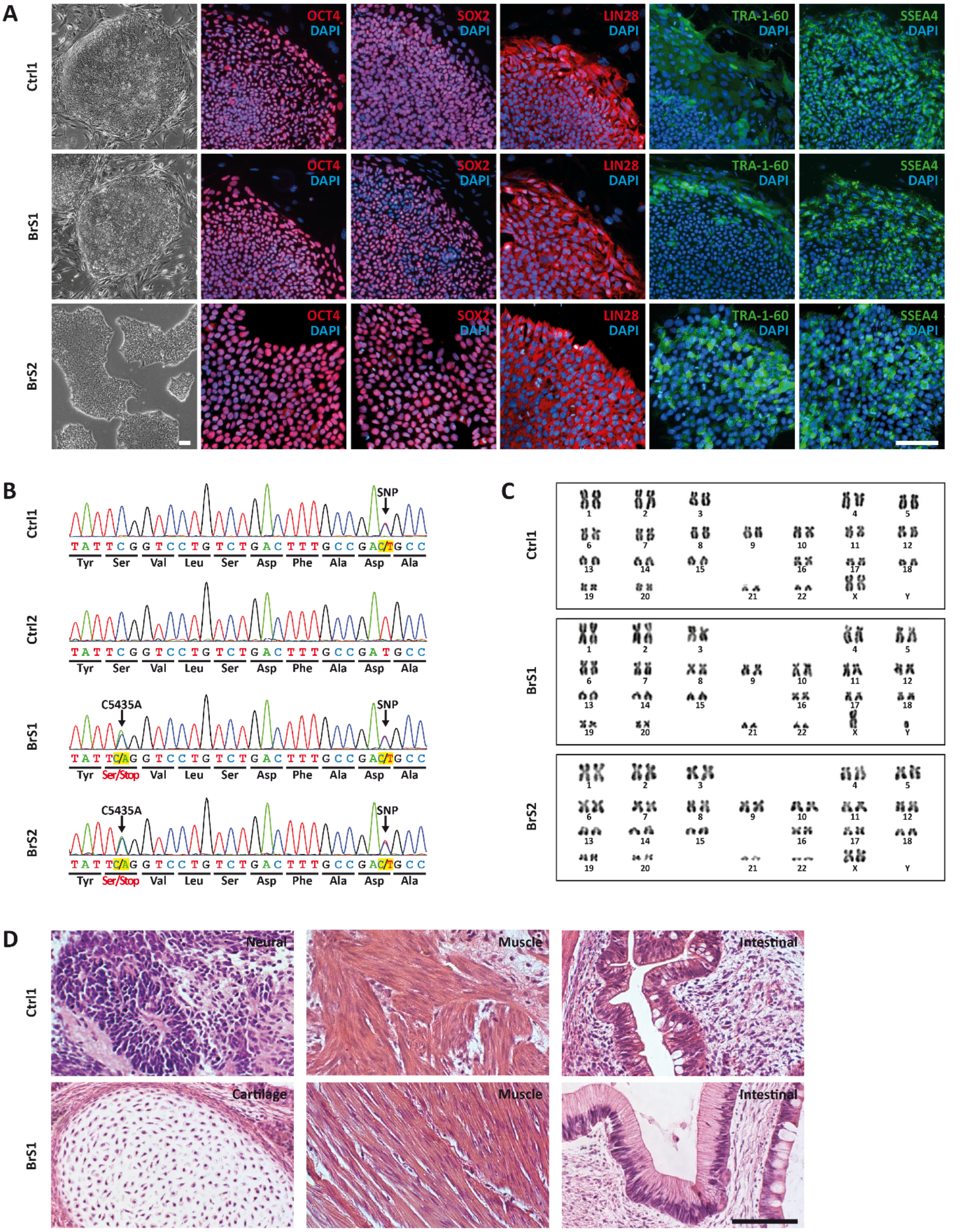

Supplementary Figure 2. Generation of iPSCs and proof of pluripotency. (**A**) Shown are morphologies of Ctrl1-, BrS1- and BrS2-iPSCs, which express pluripotency markers OCT4, SOX2, LIN28, TRA-1-60 and SSEA4. Nuclei were stained with 4',6-Diamidin-2-phenylindol (DAPI; blue). Scale bar, 200 µm. (**B**) Presence of the *SCN5A* mutation p.S1812X in BrS1- and BrS2-iPSCs and its absence in Ctrl1- and Ctrl2-iPSCs. (**C**) Normal karyotype of Ctrl1-, BrS1- and BrS2-iPSCs at passage 30. (**D**) Teratoma derived from Ctrl1- and BrS1-iPSCs comprising tissues of all three germ layers. Scale bar, 100 µm.

Fig. S3

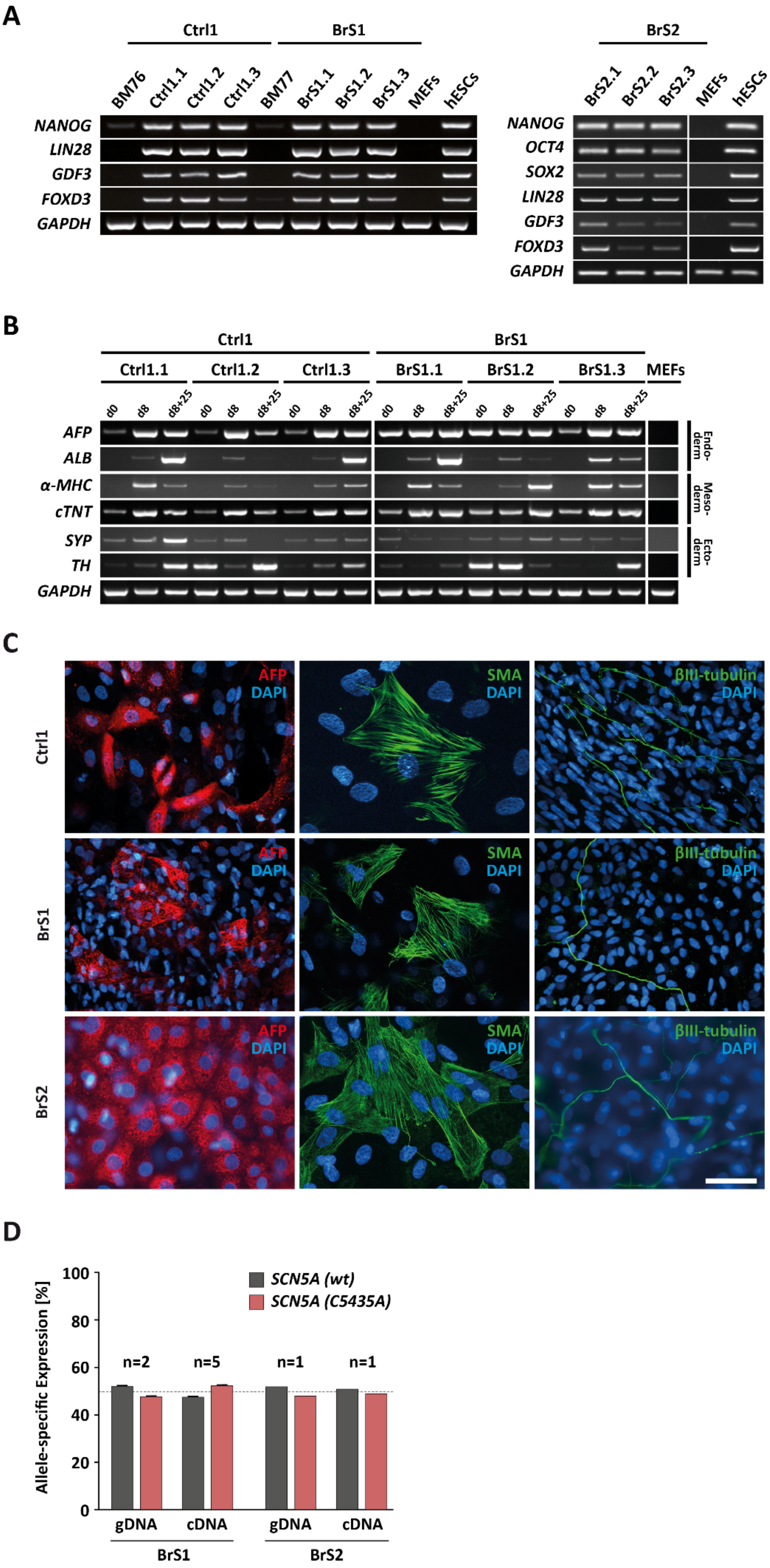
A
Ctrl1
BrS1
BM76
Ctrl1.1
Ctrl1.2
Ctrl1.3
BM77
BrS1.1
BrS1.2
BrS1.3
MEFs
hESCs
NANOG
LIN28
GDF3
FOXD3
GAPDH
BrS2
BrS2.1
BrS2.2
BrS2.3
MEFs
hESCs
NANOG
OCT4
SOX2
LIN28
GDF3
FOXD3
GAPDH
B
Ctrl1
BrS1
Ctrl1.1
Ctrl1.2
Ctrl1.3
BrS1.1
BrS1.2
BrS1.3
MEFs
d0
d8
d8+25
AFP
ALB
α-MHC
cTNT
SYP
TH
GAPDH
Endo-derm
Meso-derm
Ecto-derm
C
Ctrl1
BrS1
BrS2
AFP
DAPI
SMA
DAPI
βIII-tubulin
DAPI
D
Allele-specific Expression [%]
100
80
60
40
20
0
SCN5A (wt)
SCN5A (C5435A)
n=2
n=5
n=1
n=1
gDNA
cDNA
gDNA
cDNA
BrS1
BrS2

Supplementary Figure 3. Proof of pluripotency of generated iPSCs and expression of the *SCN5A* gene. (**A**) Shown are the endogenous expression of pluripotency-related genes *NANOG*, *LIN28*, *GDF3* and *FOXD3* in undifferentiated Ctrl1-, BrS1- and BrS2-iPSCs and their parental cells. Mouse embryonic fibroblasts (MEFs) were used as negative and human embryonic stem cells (hESCs) as positive controls. (**B**) Expression of tissue-specific genes in *in vitro* differentiated iPSCs. mRNA was isolated on day 0 (d0), 8 (d8), and 8 + 25 (d8 + 25) of differentiation. The tissue-specific genes *ALB* (albumin), *α-MHC* (α-myosin heavy chain), *TNNT2* (cardiac troponin T), and *TH* (thyroxine hydroxylase) were expressed in a developmentally controlled manner during differentiation. MEFs were used as negative control. (**C**) Immunostaining of differentiated iPSCs detecting the tissue-specific proteins α-fetoprotein (AFP, endodermal marker), α-smooth muscle actin (SMA, mesodermal marker) and class III β-tubulin (ectodermal marker). The cell nuclei were stained with DAPI. Scale bar: 50 μm. (**D**) Allele-specific expression of *SCN5A* in BrS1- and BrS2-CMs.

Fig. S4

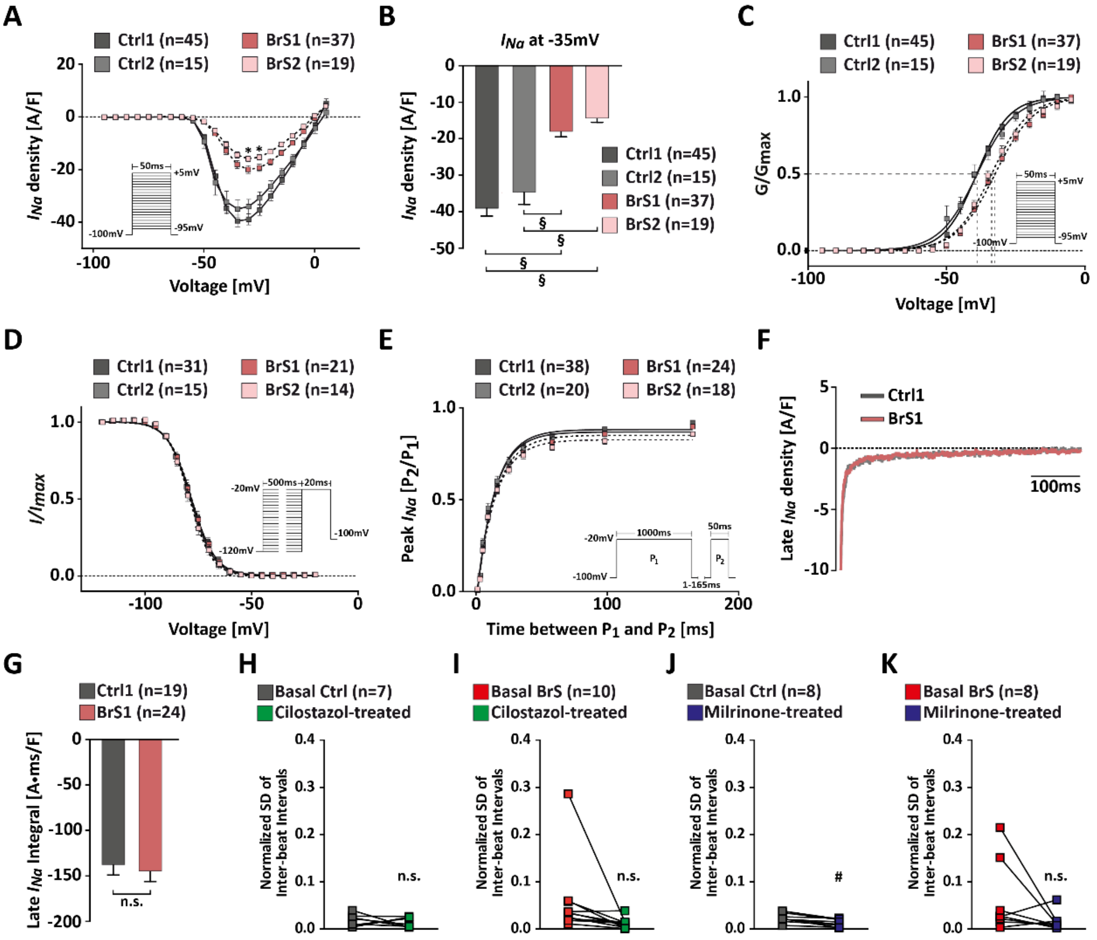

Supplementary Figure 4. Sodium channel gating properties, late I_{Na}, and the effects of cilostazol and milrinone on normalized SD of inter-beat intervals. (**A**) The average current-voltage relationship of I_{Na} in Ctrl1-, Ctrl2-, BrS1- and BrS2-CMs shows no significant difference between both control groups, but a significant difference at -30 and -25 mV between both BrS patients. Protocol is shown as inset. Data are presented as mean ± SEM. A two-way repeated measures ANOVA was used for statistical analysis: (*) P<0.05. (**B**) The peak I_{Na} densities at -35 mV are significantly reduced in both BrS patients compared to either control group. No differences were observed between two controls or two BrS. Data are presented as mean ± SEM. One way ANOVA followed by Tukey's post hoc test: (§) P<0.001. (**C**) Average of steady-state voltage dependence of activation of sodium channel in Ctrl1-, Ctrl2-, BrS1- and BrS2-CMs. Protocol is shown as inset. Data are presented as mean ± SEM. (**D**) Average of steady-state voltage dependence of inactivation of sodium channel in Ctrl1-, Ctrl2-, BrS1- and BrS2-CMs. Protocol is shown as inset. Data are presented as mean ± SEM. (**E**) Recovery from inactivation of sodium channels in Ctrl1-, Ctrl2-, BrS1- and BrS2-CMs. Protocol is shown as inset. Data are presented as mean ± SEM. (**F**) Representative late I_{Na} as depicted by original I_{Na} traces. (**G**) The averaged persistent I_{Na} densities in the interval between 50 – 450 ms in Ctrl1- and BrS1-CMs show no significant difference. Unpaired Student's t-test was used for data analysis. (**H-K**) Quantitative analysis of the normalized SD of inter-beat intervals in Ctrl-CMs (**H**) and BrS-CMs (**I**) with and without cilostazol treatment, and the normalized SD of inter-beat intervals in Ctrl-CMs (**J**) and BrS-CMs (**K**) with and without milrinone treatment. Two-tailed paired Student's t-test (H-K) were used for statistical analysis: (#) P<0.01.

Fig. S5

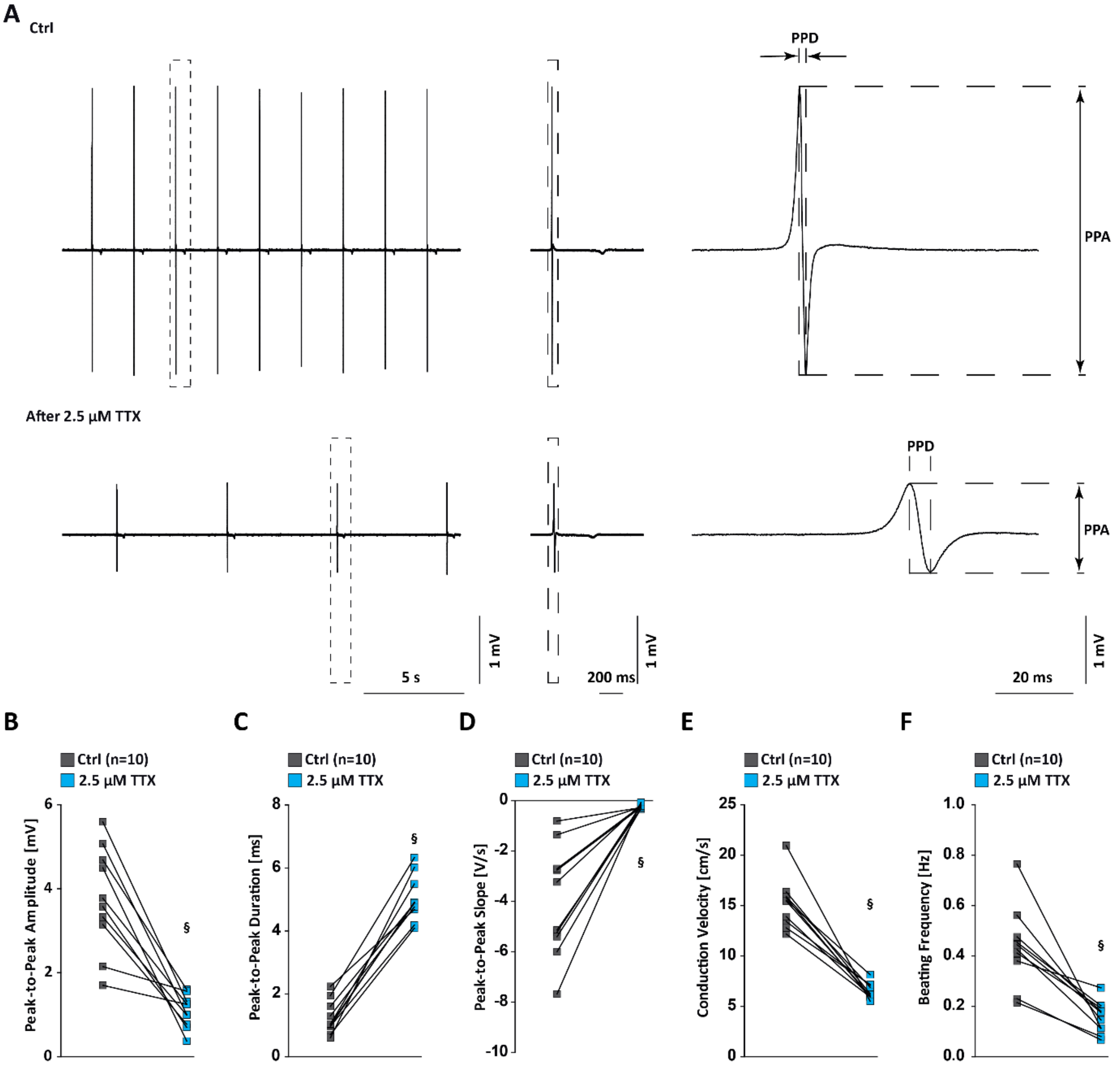

Supplementary Figure 5. The effects of TTX on field potential of Ctrl-CMs. (**A**) Original traces of field potential under basal and 2.5 µM TTX treated conditions. The differences of field potential parameters between basal and TTX treatment are shown after twice enlargements. (**B**-**F**) Panels show the analyses of peak-to-peak amplitude (**B**), peak-to-peak duration (**C**), peak-to-peak slope (**D**), conduction velocity (**E**) and beating frequency (**F**) under basal and 2.5 µM TTX treated conditions. Two-tailed paired Student's t-test was used for statistical analysis, (§) P<0.001.

Supplementary Table 1. Gating properties of the I_{Na}, I_{to} and I_{CaL} in Ctrl- and BrS-CMs.

For I_{Na}

**Parameter**	**Ctrl-CMs**	**BrS1-CMs**	**BrS2-CMs**
**Steady-state activation**			
$V_{1/2}$ [mV]	-39.1 ± 0.2	-32.4 ± 0.2§	-33.7 ± 0.3§
k_{∞} [mV]	6.1 ± 0.2	6.9 ± 0.2§	6.7 ± 0.2*
n	60	37	25
**Steady-state inactivation**			
$V_{1/2}$ [mV]	-78.3 ± 0.1	-78.1 ± 0.1	-78.5 ± 0.2
k_{∞} [mV]	5.5 ± 0.1	5.5 ± 0.1	5.2 ± 0.2
n	46	21	18
**Recovery from inactivation**			
A	0.88 ± 0.01	0.87 ± 0.01	0.83 ± 0.02*
τ_{rec} [ms]	15.2 ± 0.4	16.9 ± 0.6*	15.7± 0.5
n	58	24	25

For I_{to}

**Parameter**	**Ctrl-CMs**	**BrS-CMs**
**Steady-state inactivation**		
$V_{1/2}$ [mV]	-30.41 ± 1.6	-30.42 ± 0.72
k_{∞} [mV]	-6.76 ± 0.87	-5.55 ± 0.4
n	14	24
**Recovery from inactivation**		
A	0.96 ± 0.027	0.94 ± 0.015
Fast τ_{rec} [ms]	279.7 ± 121.1	211.9 ± 135.8
Slow τ_{rec} [ms]	2854 ± 581.7	2773 ± 462.6
n	17	16

For I_{CaL}

**Parameter**	**Ctrl-CMs**	**BrS1-CMs**	**BrS2-CMs**
**Steady-state activation**			
$V_{1/2}$ [mV]	-15.54 ± 0.38	-14.45 ± 0.84	-12.51 ± 0.50§
k_{∞} [mV]	6.370 ± 0.16	6.901 ± 0.31	6.330 ± 0.23
n	46	19	32
**Steady-state inactivation**			
$V_{1/2}$ [mV]	-39.18 ± 0.22	-36.83 ± 0.38§	-34.61 ± 0.40§
k_{∞} [mV]	5.94 ± 0.22	6.16 ± 0.34	6.33 ± 0.35
n	46	18	31
**Recovery from inactivation**			
A	0.95 ± 0.004	0.95 ± 0.006	0.96± 0.004
τ_{rec} [ms]	144.8	154.1	137.6
n	36	16	28

Voltage for half-(in)activation ($V_{1/2}$), the slope (k_{∞}), the amplitude of recovery from inactivation (A), and time constant (τ_{rec}) for development of recovery from inactivation are presented as mean ± SEM. One way ANOVA followed by Tukey's post hoc test was used for statistical analysis (*) $P<0.05$, (§) $P<0.001$.

Supplementary Table 2. Action potential characteristics in Ctrl- and BrS-CMs.

	RMP [mV]	APA [mV]	V_{max} [V/s]
**Ctrl-CMs**			
ventricular-like (n=42)	-65.9 ± 0.6	105.9 ± 1.0	21.2 ± 1.2
atrial-like (n=2)	-63.1 ± 3.0	97.4 ± 3.4	17.5 ± 2.4
nodal-like (n=3)	-54.7 ± 1.8	80.3 ± 2.7	1.7 ± 0.1
**BrS1-CMs**			
ventricular-like (n=29)	-67.9 ± 0.7	104.2 ± 1.2	13.2 ± 1.2
atrial-like (n=2)	-69.9 ± 0.2	103.2 ± 1.8	15.3 ± 6.2
nodal-like (n=3)	-53.1 ± 1.5	87.4 ± 0.6	2.3 ± 0.1
**BrS2-CMs**			
ventricular-like (n=14)	-64.9 ± 0.9	98.8 ± 1.0	7.8 ± 1.4
atrial-like (n=1)	-61.8	98.5	10.5
nodal-like (n=2)	-56.5 ± 0.4	86.9 ± 2.4	1.8 ± 0.7

Resting membrane potential (RMP), action potential amplitude (APA), maximal upstroke velocity (V_{max}).

Supplementary Table 3. Primers for pluripotency-specific, germ layer and cardiac-specific genes.

Gene	Sequence	T_A (°C)	Cycle
FOXD3 (353 bp)	for: 5′-GTG AAG CCG CCT TAC TCG TAC-3′<br>rev: 5′-CCG AAG CTC TGC ATC ATG AG-3′	61	38
GAPDH (265 bp)	for: 5′-AGA GGC AGG GAT GAT GTT CT-3′<br>rev: 5′-TCT GCT GAT GCC CCC ATG TT-3′	55	34
GDF3 (331 bp)	for: 5′-TTC GCT TTC TCC CAG ACC AAG GTT TC-3′<br>rev: 5′-TAC ATC CAG CAG GTT GAA GTG AAC AGC ACC-3′	54	32
LIN28 (410 bp)	for: 5′-AGT AAG CTG CAC ATG GAA GG-3′<br>rev: 5′-ATT GTG GCT CAA TTC TGT GC-3′	52	36
NANOG (164 bp)	for: 5′-AGT CCC AAA GGC AAA CAA CCC ACT TC-3′<br>rev: 5′-ATC TGC TGG AGG CTG AGG TAT TTC TGT CTC-3′	64	36
ALB (284 bp)	for: 5′-CCT TTG GCA CAA TGA AGT GGG TAA CC-3′<br>rev: 5′-CAG CAG TCA GCC ATT TCA CCA TAG G-3′	62	35
α-MHC (413 bp)	for: 5′-GTC ATT GCT GAA ACC GAG AAT G-3′<br>rev: 5′-GCA AAG TAC TGG ATG ACA CGC T-3′	60	35
TNNT2 (305 bp)	for: 5′-GAC AGA GCG GAA AAG TGG GA-3′<br>rev: 5′-TGA AGG AGG CCA GGC TCT AT-3′	55	35
SCN5A (408 bp)	for: 5′-TCA ACT TCC AGA CCT TCG CC-3′<br>rev: 5′-CGA TAC GGA GTG GCT CAG AC-3′	60	35
TH (215 bp)	for: 5′-GCG GTT CAT TGG GCG CAG G-3′<br>rev: 5′-CAA ACA CCT TCA CAG CTC G-3′	60	34
**Real-time PCR**			
Kv4.3 (196 bp)	for: 5′-TGT CAC CAT GAC CAC ACT GG-3′<br>rev: 5′-AAG GCG GGC CTT CTT TTG TG-3′	60	
Kv4.2 (104 bp)	for: 5′-CAC TAG GGT ATG GTG ACA TGG TG-3′<br>rev: 5′-CAC CGG AAC AGG TAG AGC A-3′	60	
Kv1.4 (280 bp)	for: 5′-CCT CCA CCT GCC AAA CCC GA-3′<br>rev: 5′-CCC TGG GAG GTA GAG AAG GTG CT-3′	60	
TNNT2 *(170 bp)*	for: 5′-AGA AGG CCA AGG AGC TGT GGC A-3′<br>rev: 5′-CCA GCG CCC GGT GAC TTT AGC-3′	60	
RPL32 (153 bp)	for: 5′-CAT CTC CTT CTC GGC ATC A-3′<br>rev: 5′-AAC CCT GTT GTC AAT GCC TC-3′	60	

Supplementary Table 4. Antibodies for pluripotency-specific, germ layer and cardiac-specific markers.

Primary	Specifications	Secondary	Specifications
**LIN28**	goat IgG, 1:300, R&D	Cy3-conjugated donkey-α-goat IgG	1:600, Jackson ImmunoResearch
**OCT4**	goat IgG, 1:40, R&D	Cy3-conjugated donkey-α-goat IgG	1:600, Jackson ImmunoResearch
**SOX2**	mouse IgG2A, 1:50, R&D	Cy3-conjugated goat-α-mouse IgG + IgM	1:300, Jackson ImmunoResearch
**SSEA-4**	mouse IgG, 1:100, Abcam	FITC-conjugated goat-α-mouse IgG	1:200, Jackson ImmunoResearch
**TRA-1-60**	mouse IgM, 1:200, Abcam	FITC-conjugated goat-α-mouse IgM	1:100, Jackson ImmunoResearch
**AFP**	rabbit IgG, 1:100, Dako	Cy3-conjugated goat-α-rabbit IgG	1:800, Jackson ImmunoResearch
**SMA**	mouse IgG2A, 1:3000, Sigma-Aldrich	Alexa 488-conjugated goat-α-mouse IgG	1:200, Thermo Fisher Scientific
**Class III β-tubulin**	mouse IgG2A, 1:2000, Covance	FITC-conjugated goat-α-mouse IgG	1:200, Thermo Fisher Scientific
**α-actinin**	mouse IgG1, 1:1000, Sigma-Aldrich	Cy3-conjugated goat-α-mouse IgG + IgM	1:300, Jackson ImmunoResearch
**cTnT**	mouse IgG1, 1:200, Thermo Fisher Scientific	Alexa 488-conjugated goat-α-mouse IgG; HRP-conjugated goat-α-mouse	1:200, Thermo Fisher Scientific; 1:2000, Cell Signaling
**Cx43**	rabbit IgG, 1:1000, Abcam	FITC-conjugated donkey-α-rabbit IgG	1:200, Jackson ImmunoResearch
**Cx43**	mouse IgG, 1:1000, Millipore	Cy3-conjugated goat-α-mouse IgG + IgM; HRP-conjugated goat-α-mouse	1:300, Jackson ImmunoResearch; 1:2000, Cell Signaling
**Na_V1.5 (493-511)**	rabbit polyclonal, 1:200, Alomone Labs	FITC-conjugated goat-α-rabbit	1:200, Jackson ImmunoResearch
**Na_V1.5 (1978-2016)**	rabbit polyclonal, 1:200, Alomone Labs	HRP-conjugated goat-α-rabbit	1:2000, Cell Signaling
**Ca_V1.2**	rabbit polyclonal, 1:200, Alomone Labs	HRP-conjugated goat-α-rabbit	1:2000, Cell Signaling
**K_V4.3**	rabbit polyclonal, 1:200, Alomone Labs	HRP-conjugated goat-α-rabbit	1:2000, Cell Signaling

Supplementary Table 5. The formula of extracellular and intracellular solutions for manual and automated patch-clamp.

		Manual patch-clamp solutions								Patchliner solutions		
Substance (mM)	Tyrode	Bath				Pipette				Seal Enhancer	Bath	Pipette
	For seal	I_{Na}	Late I_{Na}	I_{to}	I_{CaL}	I_{Na}	Late I_{Na}	I_{to}	I_{CaL}	*For seal*	I_{Na}	I_{Na}
NaCl	138	5	135	138		5	10			130	50	10
Tetramethyl ammonium chloride		135	5								90	
KCl	4			4				10		4		
$CaCl_2$	1.8	0.4	0.4	0.4	1.8	0.36	0.36			10	2	
$MgCl_2$	1	2	2	1	1	0.92	0.92	4	5	1	1	
Mg-ATP						5	5					2
Li-GTP						0.3	0.3					
CsCl		4	4			100	95		110		4	60
CsF												60
Cs-glutamate						40	40					
Glucose	10	10	10	10	10					5	5	
HEPES	10	10	10	10	10	5	5	10	10	10	10	10
Glutamic acid								120				
TEA-Cl									20			
EGTA						1	1	10	10			
Na_2ATP								2	2			
NMDG-Cl					140							
NaH_2PO_4	0.33			0.33								
Niflummic acid						0.03	0.03					
Nifedipine						0.02	0.02				0.01	
Strophanthidine						0.004	0.004					
$CdCl_2$				0.3								
pH adjustment	7.3 NaOH	7.4 NaOH	7.4 CsOH	7.3 NaOH	7.3 HCl	7.2 CsOH	7.2 CsOH	7.2 KOH	7.2 CsOH	7.4 NaOH	7.4 NaOH	7.2 CsOH

Supplementary Movie 1
The representative movie for the FP propagation of Ctrl2-CMs.

Supplementary Movie 2
The representative movie for the FP propagation of BrS1-CMs.
